# Individualized Prediction of Survival by a 10-Long Non-coding RNA-Based Prognostic Model for Patients With Breast Cancer

**DOI:** 10.3389/fonc.2020.515421

**Published:** 2020-10-19

**Authors:** Xuemei Yang, Juan Li, Yifan Wang, Peilong Li, Yinghui Zhao, Weili Duan, Abakundana Nsenga Ariston Gabriel, Yingjie Chen, Haiting Mao, Yunshan Wang, Lutao Du, Chuanxin Wang

**Affiliations:** ^1^Department of Clinical Laboratory, The Second Hospital of Shandong University, Jinan, China; ^2^Tumor Marker Detection Engineering Technology Research Center of Shandong Province, Jinan, China; ^3^Tumor Marker Detection Engineering Laboratory of Shandong Province, Jinan, China; ^4^The Clinical Research Center of Shandong Province for Clinical Laboratory, Jinan, China

**Keywords:** breast cancer, long non-coding RNA, prognosis, signature, nomogram

## Abstract

Deregulations of long non-coding RNAs (lncRNAs) have been implicated in the progression of breast cancer (BC). However, the prognostic values of those lncRNAs in BC remain elusive. This study aimed at constructing a lncRNA-based prognostic model to improve the clinical management of BC. Systematic investigation of lncRNA expression profiles and clinical data from The Cancer Genome Atlas (TCGA) database were utilized to establish a 10-lncRNA signature. The prognostic signature efficiently discriminated patients with significantly different prognosis regardless of intrinsic molecular subtypes and tumor–node–metastasis (TNM) stage. A combined model was constructed by multivariate Cox proportional hazards regression (CPHR) analysis, which combined the lncRNA-based signature with certain clinical risk factors (TNM stage, age, and human epidermal growth factor receptor 2 status). This model predicted a survival probability that closely corresponds to the actual survival probability. With respect to the entire set, the time-dependent receiver-operating characteristic curves revealed that the area under the curve of this model was the highest than any of the clinical risk factors. Moreover, functional enrichment analysis indicated that the molecular signature was mainly involved in DNA replication, which was firmly related to BC tumorigenesis. Consistent with the discovery, the knockdown of LHX1-DT, one of the 10 prognostic lncRNAs, attenuated the proliferation of BC cells *in vitro* and *in vivo*. Taken together, our study constructed a novel 10-lncRNA signature for prediction prognosis, and the signature-based model could provide new insight into accurate management of BC patients.

## Introduction

Breast cancer (BC) is one of the most commonly diagnosed malignancies among females, causing 15.4% newly identified cancers and 7.0% deaths per year in both sexes (1, 2). The overall 5-year survival rate among BC patients has improved in the past decades (3). This significant survival improvement partly benefits from the recognition of the intrinsic molecular landscape. Currently, molecular studies demonstrated that there were at least four intrinsic molecular subtypes of BC, namely luminal, human epidermal growth factor receptor 2 (HER2)-enriched, basal-like, and normal-like. The subtypes mentioned above differ in their histopathological features, biological phenotypic, and treatment sensitivities (4, 5). This diversity brings challenges in the characterization of BC while opening up new horizons for early diagnosis, therapeutic strategies determination, and prognosis prediction. Therefore, a better understanding of the molecular alterations of BC is the most important key to enhance the performance of prognosis prediction for individual people harboring BC.

With the development of large-scale sequencing and bio-information annotation, a new class of longer RNAs (>200 nt) has been recognized gradually. Such kind of RNAs lack the evident capacity of coding proteins, defined as long non-coding RNAs (lncRNAs) (6, 7). To date, lncRNAs have shown more and more importance in gene regulation at both the transcriptional and epigenetic levels (8–12). Increasing shreds of evidence have proven that lncRNAs were implicated in a variety of biological processes and have shown altered expression patterns in specific cancer types (13–16). Given the relatively stable local secondary structures and high cancer specificity, lncRNAs may act as an ideal class of biomarkers with potential applications in survival prediction (17–21).

The arrival of the big data era makes many large-scale gene expression profiles and patients’ survival data publicly available, such as The Cancer Genome Atlas (TCGA) dataset. Various useful information from TCGA can be explored with multiple statistical analyses. In this study, we repurposed the RNA expression profiles with corresponding clinical information of BC cases from the TCGA-BRCA project. The 10-lncRNA signature was identified *via* multivariate Cox proportional hazards regression (CPHR) model with forward stepwise regression. Based on the molecular signature, we further integrated it with clinical risk factors to establish a composite prognostic model. Additionally, we explored the potential biological function of the 10-lncRNA signature in BC. These results indicated that our multi−lncRNA−based model could be used as a useful prognostic predictor and guide clinical decision support for patients with BC.

## Materials and Methods

### Data Curation and Processing

RNA sequencing data including 1,109 BC cases and 113 normal cases with clinical data were collected from the TCGA-BRCA project. All data were downloaded from the GDC Data Portal, which was updated on January 31, 2019. RNA expression profiles were presented as HT-seq raw read count and annotated with Ensemble reference databases^1^. The data concerning lncRNA and mRNA expression were normalized by the “DESeq2” package in R. Meanwhile, we used this package to get differently expressed lncRNAs (DELs) and mRNAs (DEMs), setting the criteria of log_2_| fold change| >2 and adjusted *P* < 0.01.

### Definition of Prognostic LncRNAs

Two samples without complete follow-up information were removed. Univariate CPHR analysis and the Kaplan–Meier method were performed to generate the prognostic lncRNAs which were significantly related to the overall survival (OS) of BC cases. Out of 1,107 BC cases, only 718 cases had defined clinical characteristics including age, gender, T stage, N stage, M stage, tumor–node–metastasis (TNM) stage, estrogen receptor (ER) status, progesterone receptor (PR) status, HER2 status, and triple-negative breast cancer (TNBC). These 718 BC cases were randomly distributed to the training set and validation set by using a computer-generated allocation sequence in R. A multivariate Cox regression model (forward stepwise) was then used to adjust prognostic lncRNAs in the training set, which further validated with the lowest Akaike information criteria by the “MASS” package in R.

### Establishment and Validation of a LncRNA-Based Signature for Survival Prediction

The remaining prognostic lncRNAs were fitted in the multivariate CPHR analysis to calculate the coefficients for each of them in the training set. A risk score formula was constructed as follows:

$$\mathrm{Risk}\,\mathrm{Score}=\sum_{i=1}^{n}\left(\text{coeffcient}\,\left(\text{lncRNAi}\right)\times \text{expression}\,\left(\text{lncRNAi}\right)\right)$$

LncRNAi is the identifier of the inserted lncRNAs. The medium-risk score from the training set was considered as the cutoff value to divide cases into high-risk group and low-risk group. The Kaplan–Meier method and log-rank test were used to compare the OS of different patient groups. The time-dependent receiver-operating characteristic (ROC) curves were established to assess the predictive values of this risk score formula. Furthermore, stratified analysis was carried out to detect whether the lncRNA-based classifier could clearly distinguish BC cases at different risk groups within certain clinical characteristics.

### Construction of a Signature-Based Prognostic Model

Taking clinical risk factors into consideration, we performed univariate and multivariate CPHR analysis to select the independent risk factors in the total set. Based on the independent risk factors, a graphic nomogram was then constructed to predict the probability at 3- and 5-year OS by using the “rms” R package. We also integrated the independent clinical variables (age, TNM stage, HER2 status) into a new clinical model by using the “rms” R package. The ability of discrimination was evaluated by the C-index (a concordance measure analogous to the ROC curve). Calibration plots, plotted by bootstrap validation with 1,000 resamples, were used to further test the consistency of survival probability between prediction by the nomogram and actual observation.

### Functional Enrichment Analysis

Pearson correlation coefficients were calculated between the prognostic lncRNAs and DEMs based on their expression levels. DEMs with a positive correlation coefficient ≥0.4 were considered as co-expressed genes and subjected to enrichment analysis. Gene Ontology (GO) and Kyoto Encyclopedia of Genes and Genomes (KEGG) pathway analysis were performed by the “ClusterProfiler” package in R. Significant functional categories were identified, limited to KEGG pathway categories, and GO biological processes, cellular components, and molecular functions terms, using the human being whole genome as reference.

### *In Situ* Hybridization

*In situ* hybridization (ISH) arrays containing BC tissues (HBreD140Su05) and normal breast tissues (HBreD077Su01) were produced from Shanghai Outdo Biotech. *In situ* detection of LHX1-DT was performed on paraffin-embedded sections using a DIG-labeled probe (Exiqon, Denmark). Each hybridization procedure included the positive controls (U6; Exiqon, Denmark) and scrambled control RNAs. Slides were examined independently by two investigators. LHX1-DT expression was quantified by multiplying the intensity (1–3: 1, weak staining; 2, moderate staining; 3, strong staining) and extent (the percentage of positive cells on a scale of 0–4: 0, none; 1, 1–25%; 2, 26–50%; 3, 51–75%; 4, 76–100%) of staining for each tissue point using a visual grading system. The study protocol followed the ethical guidelines of the Declaration of Helsinki. This study was approved by the Research Ethics Committee of the Second Hospital of Shandong University.

### Cell Culture and Treatments

The human normal epithelial breast cell line MCF-10A and BC cell lines MCF-7, BT-549, MDA-MB-231, and MDA-MB-468 were obtained from the Cell Bank of the Chinese Academy of Sciences (Shanghai, China). MCF-7, MDA-MB-231, and MDA-MB-468 cells were cultured in DMEM (Gibco, CA, United States) and BT-549 cells were cultured in RPMI-1640 medium (Gibco, CA, United States) supplemented with 10% fetal bovine serum (Australia Origin, Gibco, Carlsbad, CA, United States) and 1% penicillin/streptomycin (Solarbio, Beijing, China), respectively. Meanwhile, MCF-10A cells were cultured in DMEM/F12 (MACGENE, Beijing, China) with 5% HS (horse serum; EVERY GREN, Hangzhou, China), 10 μg/ml insulin (MACGENE, Beijing, China), 20 ng/ml EGF (MACGENE, Beijing, China), 100 ng/ml cholera toxin (MACGENE, Beijing, China), and 0.5 μg/ml hydrocortisone (MACGENE, Beijing, China). The cells were grown in a humidified atmosphere of 5% CO_2_ at 37°C and not contaminated by mycoplasma.

### siRNA Transfection and Lentivirus Transduction

siRNA oligos targeting LHX1-DT (sense: CCUAGGUCAGAGC ACUAUUTT; antisense: AAUAGUGCUCUGACCUAGGTT) and negative control siRNA (sense: UUCUCCGAACGUGUCAC GUTT; antisense: ACGUGACACGUUCGGAGAATT) were purchased from GenePharma (Shanghai, China). MCF-7 and BT-549 cells were cultured in 60 mm dishes. Then they were transfected with 100 nM siRNA and Lipofectamine 2000 (Life Technologies) following the manufacturer’s instructions. To establish stable knockdown cell lines, specific LHX1-DT shRNA based on the corresponding sequences of LHX1-DT siRNA was produced by GenePharma (Shanghai, China). MCF-7 cells were infected with lentivirus and selected with 1 μg/ml puromycin. The selection was repeated three to four times until green fluorescent protein (GFP) was observed in all cells under a fluorescence microscope (Axio Observer, Zeiss, Oberkochen, Germany).

### RNA Extraction and Quantitative Real-Time Polymerase Chain Reaction

Total RNA was extracted using RNA fast 2000 Reagent (Fastagen, Shanghai, China). The concentration and integrity of RNA were measured by a NanoDrop spectrophotometer (Thermo Fisher Scientific, Waltham, MA, United States). Reverse transcription reactions were carried out using the PrimeScript^TM^ RT reagent kit (Takara, Dalian, China), and quantitative real-time polymerase chain reaction (qRT-PCR) was performed using TB Green^TM^ Premix Ex Taq^TM^ (Takara, Dalian, China) in the CFX-96 real-time PCR System (Bio-Rad, Shanghai, China) (details referred to the manufacturer’s instructions). The primers were synthesized as follows: LHX1-DT forward 5′-AAGAGTGACAA GGCCGTGAA-3′, LHX1-DT reverse 5′-GTAGCAGGGTGTGT ACTCCG-3′; GAPDH forward 5′-ACCCACTCCTCCACCTTT GAC-3′, GAPDH reverse 5′-TGTTGCTGTAGCCAAATTCG TT-3′. Each sample was performed in triplicate. The relative expression of target genes was calculated using the 2^–ΔΔCT^ method, while GAPDH was viewed as an internal control.

### Colony Formation Assays

After the transfection of MCF-7 and BT-549 cells, 1 × 10^3^ cells were seeded into a six-well plate (Corning, MA, United States). The clones were washed with phosphate-buffered saline and fixed in methanol for 30 min, then washed again and stained with 0.1% crystal violet for 30 min. Finally, the clones were imaged by a microscope (Axio Observer, Zeiss, Oberkochen, Germany) and quantified by ImageJ Pro Plus (version 6.0).

### 5-Ethynyl-2′-Deoxyuridine Incorporation Assays

MCF-7 and BT-549 cells were transfected with LHX1-DT siRNA and negative control siRNA. Then, cells were seeded in a 96-well plate with a density of 1 × 10^4^ cells/well. When cells were at logarithmic phase, the conditioned medium was replaced by fresh medium containing 50 μM 5-ethynyl-2′-deoxyuridine (EdU) (RiboBio, Guangzhou, China) and incubated for 3 h. The detailed steps were performed according to the manufacturer’s instructions. Finally, the images were captured using a fluorescence microscope (Axio Observer, Zeiss, Oberkochen, Germany).

### Cell Impedance Assays With the Real-Time Cell Analyzer

Cell proliferation in real-time was monitored by the xCELLigence RTCA DPlus instrument (ACEA Biosciences, San Diego, CA, United States). After transfection, MCF-7 and BT-549 cells were seeded into the wells of the E-plate 16 at 4,000 and 5,000 cells/well, respectively. The impedance signals were continuously measured every 30 min for about 72 h at 37°C. Data were recorded and analyzed using the RTCA DPlus Software (version 1.0, ACEA Biosciences, San Diego, CA, United States) and GraphPad Prism 5.0 (GraphPad Software, San Diego, CA, United States).

### *In vivo* Tumorigenesis Assays

Four-week-old female BALB/c nude mice were purchased from the Beijing Vital River Laboratory Animal Technology and housed in specific pathogen-free barrier facilities. Ten mice were randomly assigned to the control group or experimental group (five mice/group). Mice in the experimental group were subcutaneously injected with lentivirus-transduced MCF-7 cells which stably expressed LHX1-DT (7 × 10^6^ cells in 0.1 ml physiological saline) in the right flank. Meanwhile, mice in the control group were injected with 7 × 10^6^ MCF-7 cells which stably expressed scrambled. Four weeks post-injection, mice were imaged using a luminescence imaging system. At the end of imaging, all mice were euthanized by CO_2_ inhalation followed by cervical dislocation. Then, tumors were surgically dissected and weighted. Tumor volume (mm^3^) = 0.5 × width^2^ × length. All experimental procedures were conducted in conformity with the ethical standards of national and international guidelines and policies. This study was approved by the Research Ethics Committee of the Second Hospital of Shandong University.

### Statistical Analysis

The chi-square test was used to compare the relation of variables between the training set and the validation set. Univariate CPHR analysis and the Kaplan–Meier method were used to identify prognostic lncRNAs. Forward stepwise multivariate CPHR model was performed for the best-fit OS-related lncRNAs. A risk score formula was weighted by the regression coefficients derived from the above multivariate CPHR analysis. Survival analyses were performed by the Kaplan–Meier method and the log-rank test. The time-dependent ROC curve was established to assess predictive values. A composite prognostic model was constructed by the combination of molecular signature and clinical risk factors. The C-index and calibration curves were used to evaluate the discriminatory and calibration abilities of this model. All quantitative data were presented as the means ± SD of three independent experiments. Differences between two groups were analyzed by the Student’s *t* test. All statistical analyses were conducted and viewed by the R software (version 3.5.2), SPSS software (version 23.0), and GraphPad Prism 5.0 (GraphPad Software, San Diego, CA, United States). *P* < 0.05 was considered statistically significant.

## Results

### Screening of Prognostic LncRNAs

**FIGURE 1:**
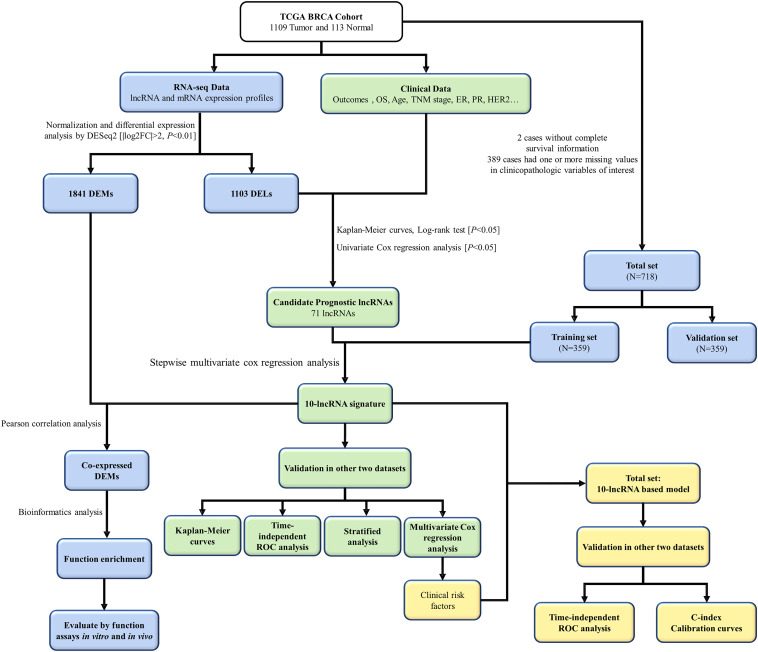
The flowchart of this study. TCGA, The Cancer Genome Atlas; lncRNA, long non-coding RNA; OS, overall survival; ER, estrogen receptor; PR, progesterone receptor; HER2, human epidermal growth factor receptor 2; DEMs, different expressed mRNAs; DELs, differently expressed lncRNAs; ROC, receiver-operating characteristic.

The flowchart of this study is shown in Figure 1. On the basis of the TCGA-BRCA project, 1,103 DELs and 1,841 DEMs were identified between 1,109 BC cases and 113 normal cases. This was achieved by setting the threshold of log_2_| fold change| >2 and adjusted *P* < 0.01 (Supplementary Figure 1). To screen the prognostic lncRNAs, we excluded two BC cases without complete survival data. Univariate CPHR analysis was performed to test the association of the expression level of DELs and the OS among 1,107 BC cases. Additionally, Kaplan–Meier curves were applied to ensure the reliability and feasibility of these prognostic lncRNAs. As it was presented in Supplementary Table 1, 71 DELs were found to be significantly correlated with OS, considered as candidate prognostic lncRNAs (*P* < 0.05, log-rank test). Moreover, 50 lncRNA higher expression levels were found to be associated with shorter survival, whereas 21 lncRNAs appeared to present an opposite trend.

### Determination of a 10-LncRNA Signature in the Training Set

To define a lncRNA-based prognostic signature, we first excluded 389 cancer cases which had insufficient clinical features such as T stage, N stage, M stage, TNM stage, ER status, PR status, and HER2 status. The remaining 718 BC cases were randomly divided into either the training set (*n* = 359) or the validation set (*n* = 359). The clinical baseline characteristics of 718 cancer cases are presented in Table 1. No significant difference was noted in the three sets of characteristics (all *P* > 0.05). Subsequently, we explored a lncRNA-based signature in the training set. Seventy-one candidate prognostic lncRNAs were filtered in multivariate CHPR analysis. After the forward stepwise model selection, we gained 10 lncRNAs (AL138789.1, AL513123.1, LINC00536, BCAR4, AC079414.1, LHX1-DT, AC006262.3, MIR3150BHG, AC105398.1, and AL133467.1) with the largest likelihood ratio and the lowest AIC value (Table 2).

**TABLE 1:** Clinical baseline characteristics of 718 breast cancer cases.

Characteristic	Training set	Validation set	Total set	*P*-value
		
	*n* = 359	*n* = 359	*n* = 718	
**Age (years)**				0.997
<65	247(68.80%)	246(68.52%)	493(68.66%)	
≥65	112(31.20%)	113(31.48%)	225(31.34%)	
**Gender**				1.000
Male	4(1.11%)	4(1.11%)	8(1.11%)	
Female	355(98.89%)	355(98.89%)	710(98.89%)	
**TNM stage**				0.584
I–II	264(73.54%)	276(76.88%)	540(75.21%)	
III–IV	95(26.46%)	83(23.12%)	178(24.79%)	
**Tumor stage**				0.716
T1–T2	306(85.24%)	298(71.87%)	604(84.12%)	
T3–T4	53(14.76%)	61(28.13%)	114(15.88%)	
**Lymph node metastasis**				0.387
Nx	2(0.56%)	3(0.83%)	5(0.69%)	
No	162(45.12%)	188(52.37%)	350(48.75%)	
Yes	195(54.32%)	168(46.80%)	363(50.56%)	
**Distant metastasis**				0.139
Mx	57(15.88%)	35(9.75%)	92(12.81%)	
No	297(82.73%)	315(87.74%)	612(85.24%)	
Yes	5(1.39%)	9(2.51%)	14(1.95%)	
**ER status**				0.560
Negative	86(23.96%)	74(20.61%)	160(22.28%)	
Positive	273(76.04%)	285(79.39%)	558(77.72%)	
**PR status**				0.085
Negative	133(37.05%)	105(29.25%)	238(33.15%)	
Positive	226(62.95%)	254(70.75%)	480(66.85%)	
**HER2 status**				0.082
Negative	266(74.09%)	291(81.06%)	557(77.58%)	
Positive	93(25.91%)	68(18.94%)	161(22.42%)	
**TNBC**				0.530
Yes	62(17.3%)	51(14.2%)	113(15.7%)	
No	297(82.7%)	308(85.8%)	605(84.3%)	

*The criteria of age referred to the World Health Organization (WHO). ER, estrogen receptor; PR, progesterone receptor; HER2, human epidermal growth factor receptor 2.*

**TABLE 2:** Ten prognostic lncRNAs significantly associated with overall survival in the training set.

Gene name	Coefficient	Type	Down-/upregulated	HR	95% CI	*P*-value
AL138789.1	0.634	Risky	Up	1.886	1.273–2.794	0.002
AL513123.1	0.488	Risky	Up	1.628	1.182–2.243	0.003
LINC00536	0.254	Risky	Up	1.289	1.014–1.639	0.038
BCAR4	0.262	Risky	Up	1.299	1.065–1.585	0.010
AC079414.1	0.825	Risky	Up	2.282	1.532–3.400	<0.01
LHX1-DT	0.253	Risky	Up	1.288	1.020–1.627	0.033
AC006262.3	1.115	Risky	Up	3.049	1.747–5.322	<0.01
MIR3150BHG	–0.779	Protective	Up	0.459	0.312–0.675	<0.01
AC105398.1	–1.414	Protective	Down	0.243	0.074–0.800	0.020
AL133467.1	–0.739	Protective	Down	0.478	0.290–0.787	0.004

*lncRNA, long non-coding RNA; HR, hazard ratio; CI, confidence interval.*

**FIGURE 2:**
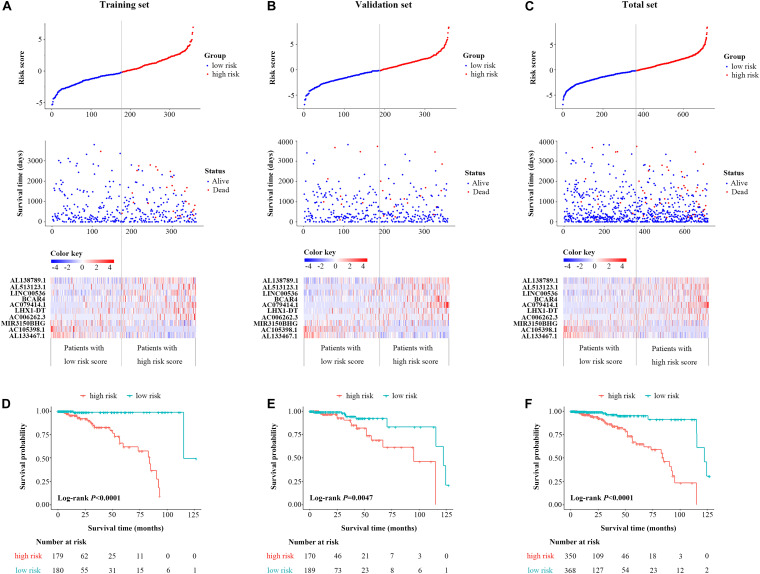
Identification and validation of a 10-lncRNA signature. The distribution of risk score, OS status, and the heatmap of the 10-lncRNA signature in the training set **(A)**, validation set **(B)**, and total set **(C)**. Kaplan–Meier curves of OS between high-risk and low-risk patients in the training set **(D)**, validation set **(E)**, and total set **(F)**. lncRNA, long non-coding RNA; OS, overall survival.

Based on the coefficients of 10 prognostic lncRNAs from multivariate CPHR analysis, we generated a risk score formula as follows: Rick Score = (0.634 × Expression_AL__138789.1_) + (0.488 × Expression_AL__513123.1_) + (0.254 × Expression_LINC00536_) + (0.262 × Expression_BCAR4_) + (0.825 × Expression_AC079414.1_) + (0.253 × Expression_LHX__1__–DT_) + (1.115 × Expression_AC__006262_._3_) + (−0.779 × Expression_MIR__3150__BHG_) + (−1.414 × Expression_AC__105398_._1_) + (−0.739 × Expression_AL__133467_._1_). The risk score for each case was calculated by corresponding expression levels of lncRNAs. These scores were ranked, and their survival statuses were plotted on a dot plot (Figure 2A, **upper**). The mortality for cases with a high-risk score was greatly higher than that with the low-risk score. The heatmap displayed the expression profiles of these 10 prognostic lncRNAs which were ranked according to the risk score (Figure 2A, below). Among the 10 lncRNAs, three lncRNAs (MIR3150BHG, AC105398.1, AL133467.1) were likely to be the protective factors, as its high expression value predicted a low risk; however, the other seven lncRNAs seemed to have the opposite potency. By using the median of risk score as the cutoff value, this signature classified BC cases of training set into the high-risk group (*n* = 179) and low-risk group (*n* = 180) (Figure 2D). The median survival time of the high-risk group was 84 months, far shorter than that of the low-risk group (median 115 months, *P* < 0.0001, log-rank test). To evaluate the predictive performance of the 10-lncRNA signature, we conducted the time-dependent ROC curves. The area under the ROC curve (AUC) was 0.886 (95% confidence interval (CI) = 0.792–0.979) at 3 years and 0.911 (95% CI = 0.835–0.987) at 5 years, which indicated a favorable discrimination performance for BC cases.

### Validation of the 10-LncRNA Signature for Survival Prediction

The molecular signature and cutoff values derived from the training set were applied to the validation set (*n* = 359) and the total set (*n* = 718). The respective distribution of the lncRNA-based risk score, OS status, and 10-lncRNA expression profiles in both sets were consistent with our findings in the training set (Figures 2B,C). For the validation set, cases in the low-risk group tended to have better survival than those in the high-risk group (median 95.1 vs. 122.3 months, *P* = 0.047, log-rank test, Figure 2E). As shown in Figure 2F, the 10-lncRNA signature also effectively picked out the high-risk and low-risk groups in the total set (median 85 vs.122 months, *P* < 0.0001, log-rank test). The AUC of the 10-lncRNA signature at 3 years was 0.574 (95% CI = 0.400–0.748) and 0.734 (95% CI = 0.618–0.849), while the AUC of the 10-lncRNA signature at 5 years was 0.650 (95% CI = 0.482–0.817), and 0.781 (95% CI = 0.688–0.0.875) for the validation set and the total set, respectively. Exhilaratingly, the predictive performance of the 10-lncRNA signature for BC patients remained superior in the validation set and the total set.

### Independence of the 10-LncRNA Signature From Conventional Clinical Risk Factors

**FIGURE 3:**
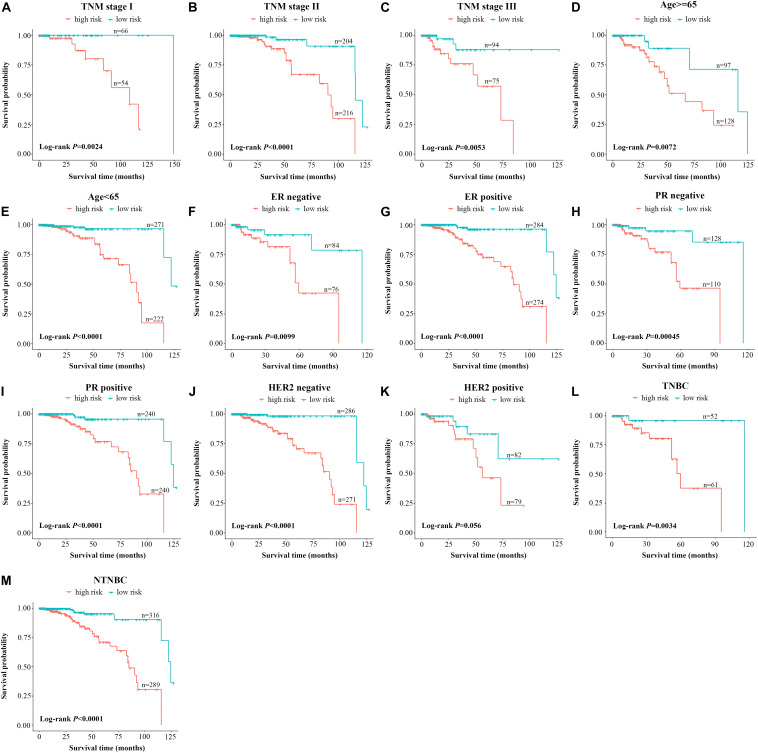
Stratified analysis of the 10-lncRNA signature for breast cancer cases by conventional clinical risk factors. Kaplan–Meier curves for breast cancer patients with TNM stage I **(A)**, II **(B)**, and III **(C)**; age ≥65 **(D)**, age <65 **(E)**; ER-negative status **(F)**, ER-positive status **(G)**, PR-negative status **(H)**, PR-positive status **(I)**, HER2-negative status **(J)**, HER2-positive status **(K)**; the triple-negative breast cancer subgroup **(L)** and the non-triple-negative breast cancer subgroup (**M**). The ticks marked on the curves represent the censored subjects. The differences between the two risk groups were accessed by the log-rank test. lncRNA, long non-coding RNA; ER, estrogen receptor; PR, progesterone receptor; HER2, human epidermal growth factor receptor 2.

Further testing was made to check whether the 10-lncRNA signature could accurately predict OS regardless of conventional clinical risk factors. Stratified analysis was performed in the total set. For each subgroup, the cases were classified as high-risk group or low-risk group according to the same cutoff value proposed above. Interestingly, the molecular signature significantly subdivided the cases into those who tend to have poorer survival or those who tend to have great survival when it was performed in the TNM stage I subgroup (*P* = 0.0024), TNM stage II subgroup (*P* < 0.0001), TNM stage III subgroup (*P* = 0.0053), age ≥65 subgroup (*P* = 0.0072), age <65 subgroup (*P* < 0.0001), ER-negative subgroup (*P* = 0.0099), ER-positive subgroup (*P* < 0.0001), PR-negative subgroup (*P* = 0.00045), PR-positive subgroup (*P* < 0.0001), triple-negative BC subgroup (*P* = 0.0034), and non-triple BC subgroup (*P* < 0.0001) (Figures 3A–I,L,M). Meanwhile, cases with low-risk scores had significantly better OS than patients with high-risk scores in the T1 subgroup, T2 subgroup, T3 subgroup, N0 subgroup, N1 subgroup, and N2 subgroup (all *P* < 0.05, log-rank test) (Supplementary Figure 2). However, as for the HER2 subgroups, this signature was significant only in the HER2-negative subgroup and did not reach the outstanding threshold in the HER2-positive subgroup (Figures 3J,K). Taken together, these results suggested that our 10-lncRNA signature can be used to identify the poorest survival cases regardless of age, TNM stage, ER status, and PR status.

### Construction of a 10-LncRNA-Based Prognostic Model to Estimate OS

**FIGURE 4:**
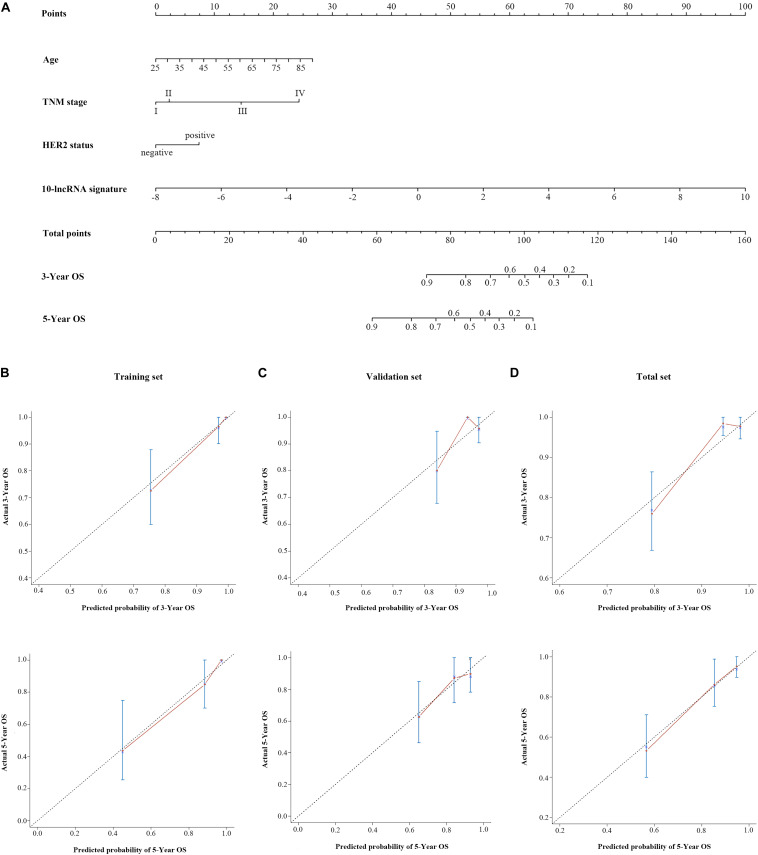
A 10-lncRNA-based prognostic model to estimate 3- and 5-year OS probability in breast cancer cases. **(A)** Nomogram to estimate OS for breast cancer cases. Instruction: Points for each variable are assigned by corresponding values from the “points” axis, and sum of the points values are located on the “total points” axis. According to a vertical line from the corresponding total points value, the 3- and 5-year OS could be easily calculated. Calibration plots of the 10-lncRNA-based prognostic model in the training set **(B)**, validation set **(C)**, and total set **(D)**. The 45° dotted line represents a perfect prediction, and the red lines represent the predictive performance of the nomogram. lncRNA, long non-coding RNA; HER2, human epidermal growth factor receptor 2; OS, overall survival.

To assist in clinical decision-making, we tried to construct a 10-lncRNA-based prognostic model that reduced statistical predictive model into a numerical estimate of the probability for OS. The univariable and multivariable CPHR analyses were performed in the total set. Given the multicollinearity among covariates, we further excluded tumor stage, lymph node metastasis, and distant metastasis (22). Table 3 shows the remaining independent variables, including age, TNM stage, HER2 status, and the 10-lncRNA signature. Hence, an innovative prognostic model, integrating both the 10-lncRNA signature and three clinical risk factors, was constructed to calculate the probability of OS (Figure 4A). This model illustrated the 10-lncRNA signature as the largest contributor to survival prediction, followed by age, TNM stage, and HER2 status, which showed a moderate effect on prediction. According to user-friendly interfaces, we could easily obtain the 3- and 5-year OS probabilities corresponding to the total score which sums up the point for each variable.

**TABLE 3:** Univariate and multivariate CPHR analyses of the 10-lncRNA signature and clinical factors with OS in total set.

Characteristic	Univariate analysis		Multivariate analysis	
	HR (95% CI)	*P*-value	HR (95% CI)	*P*-value
Age (≥65 vs. <65)	2.694 (1.554–4.669)	**> 0.001**	2.838 (1.604–5.022)	**> 0.001**
Gender (male vs. female)	1.094E-07 (0–inf)	0.997		
TNM stage (III–IV vs. I–II)	2.568 (1.452–4.541)	**0.001**	3.258 (1.768–6.002)	**> 0.001**
Tumor stage (T3–T4 vs. T1–T2)	1.700 (0.889–3.252)	0.109		
Lymph node metastasis (yes vs. no)	1.743 (0.958–3.172)	0.069		
Distant metastasis (yes vs. no)	4.739 (1.685–13.323)	**0.003**		
ER status (positive vs. negative)	0.500 (0.280–0.893)	**0.019**	0.792 (0.288–2.174)	0.650
PR status (positive vs. negative)	0.554 (0.317–0.970)	**0.039**	0.457 (0.170–1.231)	0.122
HER2 status (positive vs. negative)	2.125 (1.184–3.816)	**0.012**	1.982 (1.070–3.669)	**0.030**
10-LncRNA signature	1.436 (1.274–1.618)	**> 0.001**	1.477 (1.291–1.689)	**> 0.001**

*Bold values indicate statistical significance (*P* < 0.05). CPHR, Cox proportional hazards regression; lncRNA, long non-coding RNA; OS, overall survival; HR, hazard ratio; CI, confidence interval; ER, estrogen receptor; PR, progesterone receptor; HER2, human epidermal growth factor receptor 2.*

**FIGURE 5:**
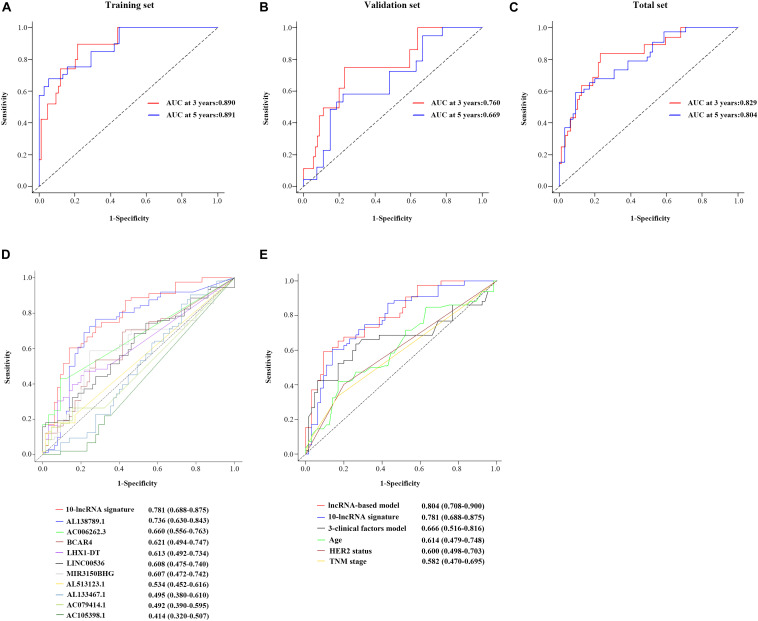
Performance evaluation of the 10-lncRNA-based prognostic model. Time-dependent ROC curves of the 10-lncRNA-based prognostic model in the training set **(A)**, validation set **(B)**, and total set **(C)**. Comparison of the prognostic accuracy at 5 years by time-dependent ROC curves in the 10-lncRNA signature with single lncRNA **(D)** and the 10-lncRNA-based prognostic model with 10-lncRNA signature, 3-clinical factors model, age, HER2 status and TNM stage **(E)**. lncRNA, long non-coding RNA; ROC, receiver-operating characteristic; AUC, the area under the ROC curve; HER2, human epidermal growth factor receptor 2.

Figure 4D shows the calibration plots of the prognostic model for the 3- and 5-year OS probabilities in the total set, which exhibited optimal agreement between prediction by nomogram and actual observation. The ability of discrimination was assessed by C-index, which quantified the concordance between predicted OS and observed OS by internal validation using 1,000 bootstrap resamples. The C-index of the 10-lncRNA signature-based model was up to 0.851 (95% CI = 0.801–0.901) in the total set. Additionally, we applied the time-dependent ROC curves to assess the predictive performance of this model. As shown in Figure 5C, the AUC vales of the combined model at 3 and 5 years were 0.829 (95% CI = 0.734–0.923) and 0.804 (95% CI = 0.708–0.900), respectively. The validity of the prognostic model was further confirmed with calibration plots and C-index in the training set and validation set. Calibration plots exhibited that the model also had a good accuracy compared with the ideal model both in the training set and validation set (Figures 4B,C). The C-index of the model was 0.891 (95% CI = 0.843–0.939) and 0.813 (95% CI = 0.719–0.907) in the two datasets. The AUC values of the model at 3 years were 0.890 (95% CI = 0.801–0.978, the training set) and 0.760 (95% CI = 0.597–0.923, the validation set), which at 5 years were 0.891 (95% CI = 0.790–0.992, training set) and 0.669 (95% CI = 0.498–0.840, validation set) (Figures 5A,B).

### Comparison With Other Clinical Prognostic Factors

By using the time-dependent ROC curves, we assessed and compared the predictive sensitivity and specificity of both the 10-lncRNA signature with a single lncRNA and the 10-lncRNA-based prognostic model with other prognostic factors. As presented in Figure 5D, the AUC of the 10-lncRNA signature at 5 years was 0.781 (95% CI = 0.688–0.875), which is generally higher compared with that of single lncRNA. Furthermore, we found that the 10-lncRNA signature had a significant OS prediction ability compared to clinical prognostic factors such as TNM stage (0.781 vs. 0.582, *P* = 0.013) and HER2 status (0.781 vs. 0.600, *P* = 0.015) (Figure 5E). In addition, the AUC of the combined prognostic model was equivalent to the 10-lncRNA signature (0.804 vs. 0.781, *P* = 0.636), but it was significantly higher than TNM stage (0.804 vs. 0.582, *P* < 0.001), HER2 status (0.804 vs. 0.600, *P* < 0.001), and age (0.804 vs. 0.614, *P* = 0.0012) (Figure 5E). Notably, the AUC of the signature-based prognostic model was also higher than the three-clinical factors model (0.804 vs. 0.666, *P* = 0.015) (Figure 5E). Taken together, the 10-lncRNA signature and the combined prognostic model had significantly better predictive performance than age, TNM stage, HER2 status, and even the three-clinical factors model.

### Functional Enrichment Analysis of the 10-LncRNA Signature

**FIGURE 6:**
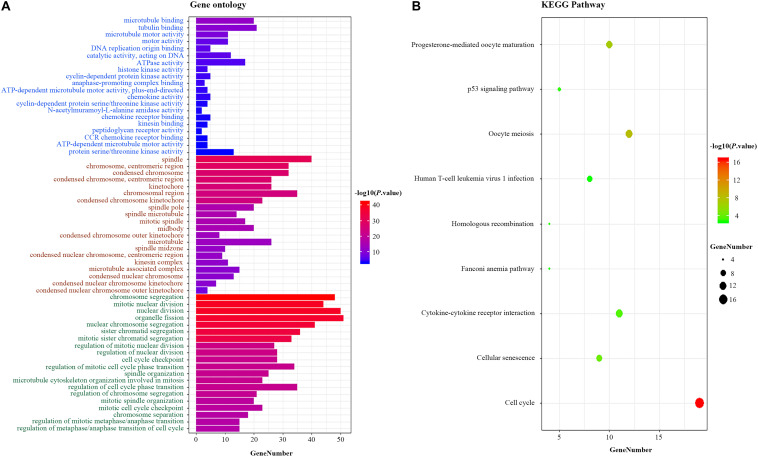
Functional enrichment analysis for the 10-lncRNA signature. **(A)** GO enrichment analysis. Blue, brown, and green words represent the GO terms in molecular functions, cellular components, and biological processes, respectively. **(B)** KEGG enrichment analysis. The *x*-axis and *y*-axis indicate the number of genes and the GO terms/KEGG pathway names, respectively. The color represents the *P*-value. lncRNA, long non-coding RNA; GO, Gene Ontology; KEGG, Kyoto Encyclopedia of Genes and Genomes.

We explored the potential function of 10 prognostic lncRNAs in the signature. By using Pearson correlation analysis between the expression levels of 10 lncRNAs and those of DEMs, 206 DEMs were identified as meaningful co-expressed genes, which correlated with at least one of the 10 lncRNAs (Pearson coefficient ≥0.4). Enrichment analysis revealed that these co-expressed DEMs were significantly enriched in 342 GO terms in biological processes, 57 GO terms in cellular components, 23 GO terms in molecular functions, and 9 KEGG pathways (Supplementary Table 2). These GO terms were found mainly to be associated with DNA replication origin binding and chromosome centromeric region. The KEGG pathways analysis showed that the co-expressed genes were involved in p53 signaling pathway, oocyte meiosis, and homologous recombination (Figures 6A,B), which were closely associated with the tumorigenesis and development of BC as described in previous studies (23–26).

### LHX1-DT, One of the 10 Prognostic LncRNAs, Enhances the Proliferation of BC Cells

**FIGURE 7:**
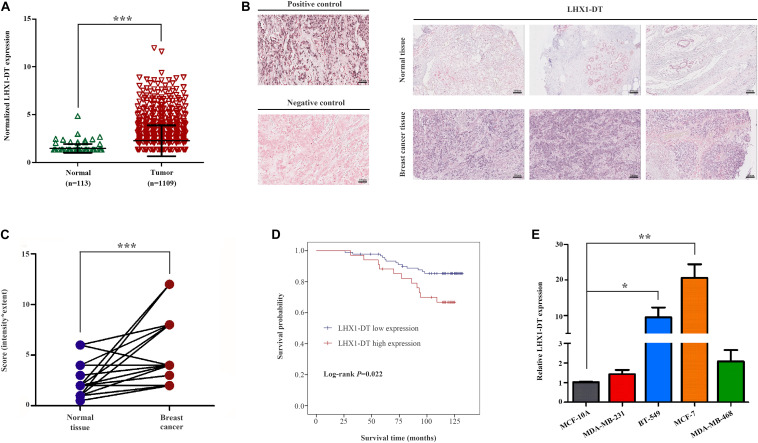
LHX1-DT was upregulated and significantly correlated with the survival of breast cancer patients. **(A)** Relative expression levels of LHX1-DT in the TCGA-BRCA project. **(B)** Representative images of LHX1-DT expression in breast cancer tissues and normal breast tissues using ISH analysis. Paraffin-embedded tissue sections were stained using specific probe for LHX1-DT in purple–blue. (Positive control, U6 snRNA; negative control, scrambled control). Scale bars, 100 μm (black). **(C)** Statistical analysis of LHX1-DT expression in 55 paired breast cancer tissues and normal breast tissues. The *y*-axis indicates the product of its staining intensity and extent of LHX1-DT. Paired Student’s *t*-test. **(D)** Kaplan–Meier survival curves analysis of OS in breast cancer patients with high or low expression of LHX1-DT. The median of LHX1-DT expression level was used as the cutoff value. Log-rank test. **(E)** The expression level of LHX1-DT is detected by qRT-PCR in MCF-7, BT-549, MDA-MB-231, MDA-MB-468, and MCF-10A cells. Results were presented as the mean ± SD. Three independent experiments, Student’s *t*-test. *P < 0.05, **P < 0.01, and ***P < 0.001. TCGA, The Cancer Genome Atlas; ISH, *in situ* hybridization; OS, overall survival; qRT-PCR, quantitative real-time polymerase chain reaction.

**FIGURE 8:**
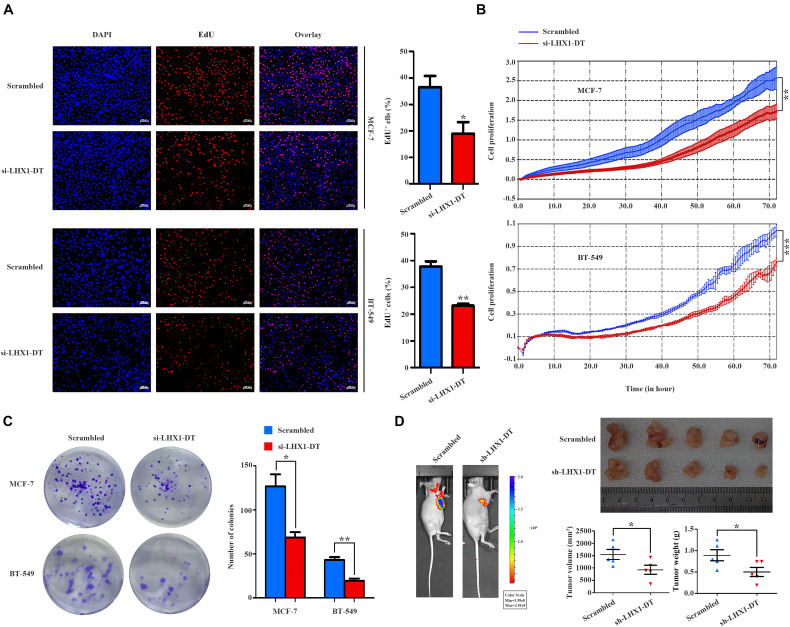
LHX1-DT promotes the cell proliferation of breast cancer cells. **(A)** EdU incorporation assays of the cell population in the S phase (left) and histogram analysis of EdU-positive cell counts are shown (right). Blue color represented the nucleus, and red color represented EdU-positive cells. Scale bars, 100 μm (white). Three independent experiments, Student’s *t*-test. **(B)** Cell impedance assays were performed with the xCELLigence RTCA DPlus instrument to monitor the cell growth dynamics of si-LHX1-DT-transfected or negative control siRNA-transfected breast cancer cells. Three independent experiments, Student’s *t* test. **(C)** Colony formation assays were performed to detect the proliferation of si-LHX1-DT-transfected or negative control siRNA-transfected breast cancer cells. Three independent experiments, Student’s *t* test. **(D)** Effect of LHX1-DT on breast cancer tumorigenesis *in vivo*. Subcutaneous xenograft assay of stable LHX1-DT knockdown MCF-7 cells or scrambled cells (7 × 10^6^ cells) in nude mice. Representative luminescence images were shown (left) (*n* = 5, per group). Effect of LHX1-DT on subcutaneous xenograft growth in mice (right). The curves of volume and weight of tumors were shown (*n* = 5, per group). Results were presented as the mean ± SD. Student’s *t*-test. *P < 0.05, **P < 0.01, and ***P < 0.001. RTCA, real-time cell analyzer.

Given the fold change and *P*-value of the 10 prognostic lncRNAs, LHX1-DT was selected and subjected to further clinical and functional assays (Supplementary Table 3). As it was shown in Figure 7A, LHX1-DT has been seen to be upregulated in BC cases based on the analyses done on the TCGA dataset. To test whether LHX1-DT upregulation was related to poor prognosis of BC patients, the expression level of LHX1-DT was evaluated by ISH in another cohort (Figure 7B and Supplementary Table 4). Results showed that the expression level of LHX1-DT was higher in BC tissues compared with paired normal breast tissues (Figure 7C and Supplementary Table 5). Meanwhile, high expression of LHX1-DT was significantly associated with shorter survival (Figure 7D). Similarly, LHX1-DT was also upregulated in cultured BC cell lines (MCF-7, BT-549, MDA-MB-231, and MDA-MB-468) compared to epithelial breast cell line MCF-10A (Figure 7E). We chose MCF-7 and BT-549 cells for further studies because of their highest expression compared with MCF-10A. The knockdown efficiency is shown in Supplementary Figure 3. The EdU incorporation assay detected a significant decrease in the proliferation of MCF-7 and BT-549 cells after silencing of LHX1-DT (Figure 8A). In parallel, both real-time cell impedance assay with RTCA and colony formation assay were performed in MCF-7 and BT-549 cells, and the results were consistent with the results above (Figures 8B,C). Then, we explored the effect of LHX1-DT in BC tumorigenesis *in vivo*. Stabled LHX1-DT-silenced or control MCF-7 cells were subcutaneously injected into female BALB/c nude mice. After 4 weeks of injection, suppression of LHX1-DT produced a significant decrease in the weight and volume of xenograft (Figure 8D). Collectively, these data strongly suggested that LHXI-DT was required for the proliferation of BC cells *in vitro* and targeting LHX1-DT could inhibit tumorigenesis and tumor growth of BC cells *in vivo*.

## Discussion

In this study, we presented a brief overview of the distinct expression patterns of lncRNAs in BC and identified a 10-lncRNA signature carrying prognostic information for BC cases. Moreover, integrated with clinical risk factors, a lncRNA signature-based prognostic model was constructed to predict individual survival probabilities. Additionally, we sought to explore the potential function of the molecular signature by GO and KEGG pathway enrichment analysis and preliminarily studied their biological functions *in vitro* and *in vivo.*

Originally considered as transcriptional noise, lncRNAs are now viewed as key molecules which contributed to various mechanisms of cancer biology. With the development of high-throughput deep sequencing, over 118,770 human lncRNAs that constitute the human genome have been identified and annotated and these continue to increase (27). Recent studies have reported that lncRNAs exhibited highly distinct expression patterns in specific diseases and even in their particular developmental stage. Therefore, lncRNAs are considered as one of the most promising biomarkers and therapeutic targets (28–32). Notably, BC is a highly heterogeneous disease with multiple molecular features. High-throughput genomic analysis is becoming a hit focus in the clinical management of BC. Increasing multigene signatures have been developed for predicting outcomes and assisting therapeutic strategies. The 21-gene Oncotype Dx assay, the Amsterdam 70-gene MammaPrint signature, and the PAM50 are most commonly used in clinical practice (33–37). However, most of these studies only focused on the application of gene expression profiles, ignoring the value of traditional clinical factors such as TNM stage, ER status, PR status, and HER2 status.

Given the genomic heterogeneity of BC, we mined the lncRNA expression profiles and survival data between BC cases and normal cases from the TCGA database and further constructed a 10-lncRNA signature for prognosis prediction. This 10-lncRNA signature could accurately classify BC cases into different risk groups. Cases in the high-risk group had significantly worse survival than those in the low-risk group. The application of reported multiple gene classifiers formerly was often limited by low reproducibility in clinical use (38–42). Our 10-lncRNA signature has a robust ability to classify patients, which was successfully validated in the validation set and total set. Besides, the AUC of the 10-lncRNA signature was the highest compared with the one for the TNM stage and age, indicating that the signature was efficient and sensitive to predict OS.

It has been widely reported that clinical factors, such as TNM, have been used most frequently and are important for diagnosis and prognosis evaluation of BC patients (43, 44). Here, we assessed the prognostic ability of conventional clinical risk factors. Three clinical factors (TNM stage, age, HER2 status) and the 10-lncRNA signature showed independence and were integrated into a combined prognostic model. According to user-friendly interfaces, clinicians could easily get the probability of 3- and 5-year OS for each BC patient. In brief, the score of each characteristic could be taken from the “points” axis. The total scores are tallied and located on the “total points” axis. The 3- or 5-year survival probabilities could be calculated by drawing a vertical line down to the corresponding axis. Bootstrap validation with 1,000 resamples suggested that the 10-lncRNA-based prognostic model had great accuracy in all of the three sets. Notably, variables in the model were relatively easily acquired, indicating that this model has the potential to be widely used in clinical practice.

One of the most attractive features of biomarkers is accurate prognosis for patients with malignant disease, which helps to stratify patients into different risk groups and to choose the most effective treatment. In the study, our molecular signature could successfully classify BC patients into different risk groups regardless of age, TNM stage, and ER and PR status. Additionally, we found that its classification efficiency was still significant in the triple-negative BC subgroup (*P* = 0.0034) and non-triple BC subgroup (*P* < 0.0001). Interestingly, the stratification of the HER2-negative subgroup showed significance in outcome; nevertheless, no clear and significant separation was observed in the HER2-positive subgroup. These results suggested that the 10-lncRNA signature could predict OS regardless of conventional molecular features and clinical factors; however, it requires further modifications to optimize its prognostic performance before adoption into clinical setting.

The newly developed 10-lncRNA signature contained two known lncRNAs (BCAR4 and LINC00536). BCAR4 (BC antiestrogen resistance 4) originally was found to participate in the development of resistance to antiestrogens in BC, whose expression is associated with proliferation, metastasis, and invasion of multiple tumors (45–48). LINC00536, a newly identified lncRNA by Nakajima in 2014, was found to be highly expressed in bladder cancer and promoted the tumorigenesis and development of bladder cancer partly by modulating the Wnt3a/β-catenin signaling (49). However, most of the 10 lncRNAs in our prognostic signature have been poorly investigated. In terms of potential function, we performed functional enrichment analysis based on the positively co-expressed DEMs with these 10 lncRNAs. The results represented that the 10 prognostic lncRNAs were possible to participate in cell proliferation. Given the truth of our inference, we selected LHX1-DT, an upregulated lncRNA with optimal log_2_| fold change| and *P*-value, to be subjected to clinical and functional assays. The results of ISH proved that LHX1-DT was upregulated in BC and significantly associated with the survival of BC patients. Downregulation of LHX1-DT markedly suppresses cell growth in cultured BC cells and xenograft mouse model which was consistent with the results of the functional prediction above. We also tried to investigate how the LHX1-DT participates in the development of BC. According to cis/trans analysis, we found that AATF and LHX1 are located above or beyond the Flank10kb with LHX1-DT and might be the target protein-coding RNAs. Unfortunately, there were none of the target mRNAs in trans. On the other hand, we searched the StarBase database to analyze the interaction between LHX1-DT and mRNAs and found that USP11, RNPEPL1, JPT2, DNASE2, and NR6A1 might be related to LHX1-DT. We focus on making use of the special lncRNA expression profiles and conventional clinical risk factors to predict the prognosis of BC patients. Therefore, more comprehensive studies about molecular mechanism will remain to be lucubrated in the future.

Our study has several limitations. First of all, to validate the performance of the 10-lncRNA signature-based prognostic model, we mainly performed internal validation with 1,000 bootstrap resamples. Although the model demonstrated a robust performance in discrimination and calibration, it did not externally validate other independent cohorts and this might result in overfitting. So, multicenter and large-scale studies should be performed to validate this predictive value before application in routine clinical practice. Second, the present study is mainly on the foundation of the TCGA-BRCA project, in which the clinical information of BC is not complete such as the Ki-67 index and radiotherapy. Third, the generalizability of the 10-lncRNA signature and signature-based prognostic model is another limitation of our study. The signature and prognostic models were generated and evaluated in the TCGA-BRCA project. BC cases enrolled mainly came from the Caucasian race, so the prognostic value for other races was questionable, and further studies with other racial cohorts are still necessarily needed.

Taken together, this study revealed a powerful 10-lncRNA signature-based prognostic model by probing and integrating currently available datasets. The newly developed 10-lncRNAs signature could accurately predict outcomes for BC patients, as well as specific characteristics of subgroups. After integrated with clinical risk factors, a combined 10-lncRNA-based prognostic model was constructed for generating a specific probability of 3- and 5-year OS for BC patients. Furthermore, we inferred and initially confirmed that the 10 prognostic lncRNAs were closely associated with the cell proliferation of BC cells. Further investigations need to be performed with regard to the validation of our findings and the functional explanation of these prognostic lncRNAs.

## Data Availability Statement

Publicly available datasets were analyzed in this study. The dataset analyzed during the current study is available in TCGA (http://cancergenome.nih.gov/), all data were downloaded from the GDC Data Portal (https://portal.gdc.cancer.gov), which is updated to January 31, 2019.

## Ethics Statement

The studies involving human participants were reviewed and approved by the Research Ethics Committee of the Second Hospital of Shandong University. Written informed consent for participation was not required for this study in accordance with the national legislation and the institutional requirements. The animal study was reviewed and approved by the Research Ethics Committee of the Second Hospital of Shandong University.

## Author Contributions

XY and JL designed this research, analyzed the data, and performed the experiments. YW took part in drafting the manuscript. PL, YZ, WD, and ANAG provided technical support. HM, YC, and YW critically revised the manuscript. LD and CW initiated, organized, and supervised the study. All authors have read and approved the final version of the manuscript.

## Conflict of Interest

The authors declare that the research was conducted in the absence of any commercial or financial relationships that could be construed as a potential conflict of interest.

## Footnotes

1 ftp://ftp.ensembl.org/pub/release-93/gtf/homo_sapiens

## Supplementary Material

Supplementary Figure 1 Volcano plots and heatmaps of 1103 DELs and 1841 DEMs in breast cancer cases. **(A,B)** Volcano plot and heatmaps of 1103 DELs in breast cancer cases. **(C,D)** Volcano plot and heatmaps of 1841 DEMs in breast cancer cases. Blue color indicates a down-regulated expression, and the red color represents up-regulated expression. Green nodes are candidate prognostic lncRNAs. lncRNA, long non-coding RNA; DELs, differently expressed lncRNAs; DEMs, different expressed mRNAs.

Supplementary Figure 2 Stratified analysis of the 10-lncRNA signature for breast cancer cases by clinicopathological risk factors. Kaplan-Meier curves for breast cancer patients with T1 **(A)**, T2 **(B)**, T3 **(C)**, N0 **(D)**, N1 **(E)**, N2 **(F)**, and N3 **(G)**. The ticks marked on the curves represent the censored subjects. The differences between the two risk groups were accessed by the log-rank test.

Supplementary Figure 3 The efficiency knockdown of the expression levels of LHX1-DT. qRT-PCR detection of LHX1-DT expression in MCF-7 and BT-549 cells transfected with scrambled or si-LHX1-DT. Three independent experiments, Student’s test. qRT-PCR, quantitative real-time polymerase chain reaction.

Supplementary Table 1 Candidate prognostic lncRNAs in the TCGA-BRCA Project.

Supplementary Table 2 Enrichment analysis of GO terms for positively co-expressed DEMs correlated with prognostic lncRNAs.

Supplementary Table 3 The summary of 10 prognostic lncRNAs.

Supplementary Table 4 Clinical baseline characteristics of breast cancer patients *in situ* hybridization analysis.

Supplementary Table 5 The quantification results of *in situ* hybridization analysis.

## Abbreviations

AUC: area under the ROC curve
BC: breast cancer
CI: confidence interval
CPHR: Cox proportional hazards regression
DELs: differently expressed lncRNAs
DEMs: different expressed mRNAs
ER: estrogen receptor
GO: Gene Ontology
HER2: human epidermal growth factor receptor 2
ISH: *in situ* hybridization
KEGG: Kyoto Encyclopedia of Genes and Genomes
lncRNA: long non-coding RNA
OS: overall survival
PR: progesterone receptor
ROC: receiver operating characteristic
RTCA: real-time cell analyzer
RT-qPCR: quantitative real-time polymerase chain reaction
TCGA: The Cancer Genome Atlas.
