# Supplementary figures and images for: Individualized Prediction of Survival by a 10-Long Non-coding RNA-Based Prognostic Model for Patients With Breast Cancer
Source: Front Oncol. 2020 Oct 19;10:515421. doi: 10.3389/fonc.2020.515421 (PMC7604500; doi:10.3389/fonc.2020.515421)

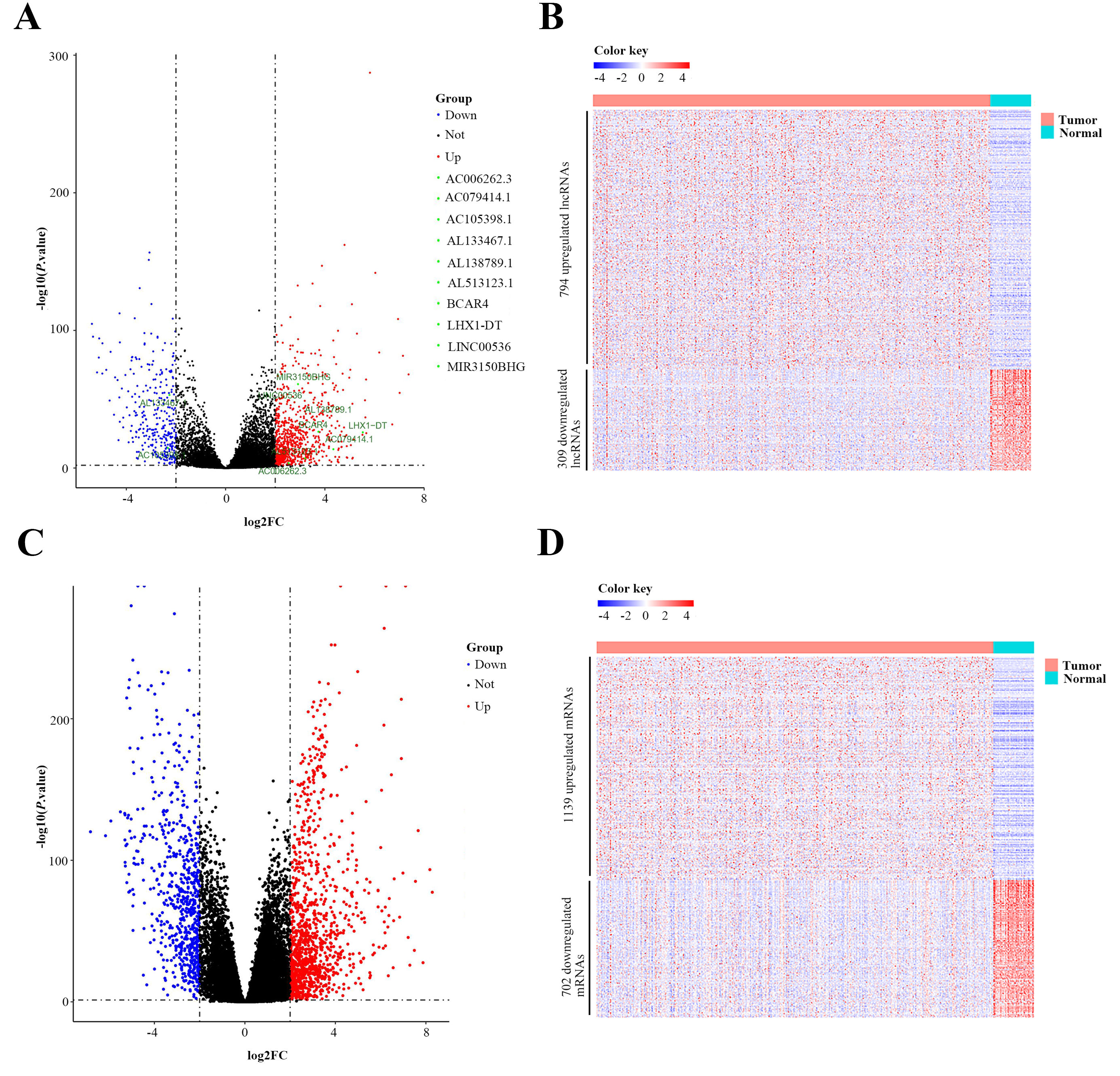

Supplement: Supplementary Figure 1 — Volcano plots and heatmaps of 1103 DELs and 1841 DEMs in breast cancer cases. (A,B) Volcano plot and heatmaps of 1103 DELs in breast cancer cases. (C,D) Volcano plot and heatmaps of 1841 DEMs in breast cancer cases. Blue color indicates a down-regulated expression, and the red color represents up-regulated expression. Green nodes are candidate prognostic lncRNAs. lncRNA, long non-coding RNA; DELs, differently expressed lncRNAs; DEMs, different expressed mRNAs. [file Image_1.TIF]

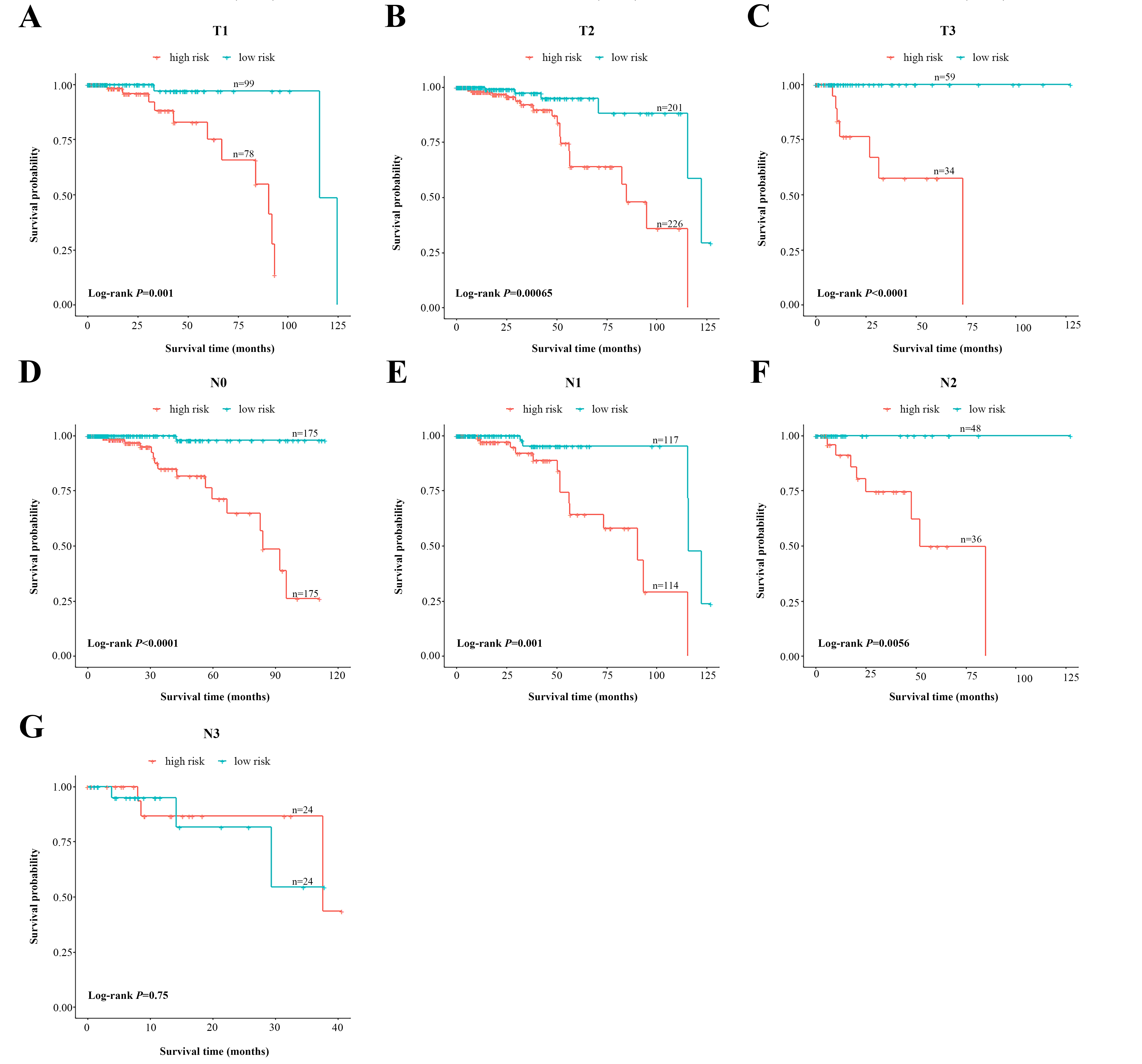

Supplement: Supplementary Figure 2 — Stratified analysis of the 10-lncRNA signature for breast cancer cases by clinicopathological risk factors. Kaplan-Meier curves for breast cancer patients with T1 (A), T2 (B), T3 (C), N0 (D), N1 (E), N2 (F), and N3 (G). The ticks marked on the curves represent the censored subjects. The differences between the two risk groups were accessed by the log-rank test. [file Image_2.TIF]

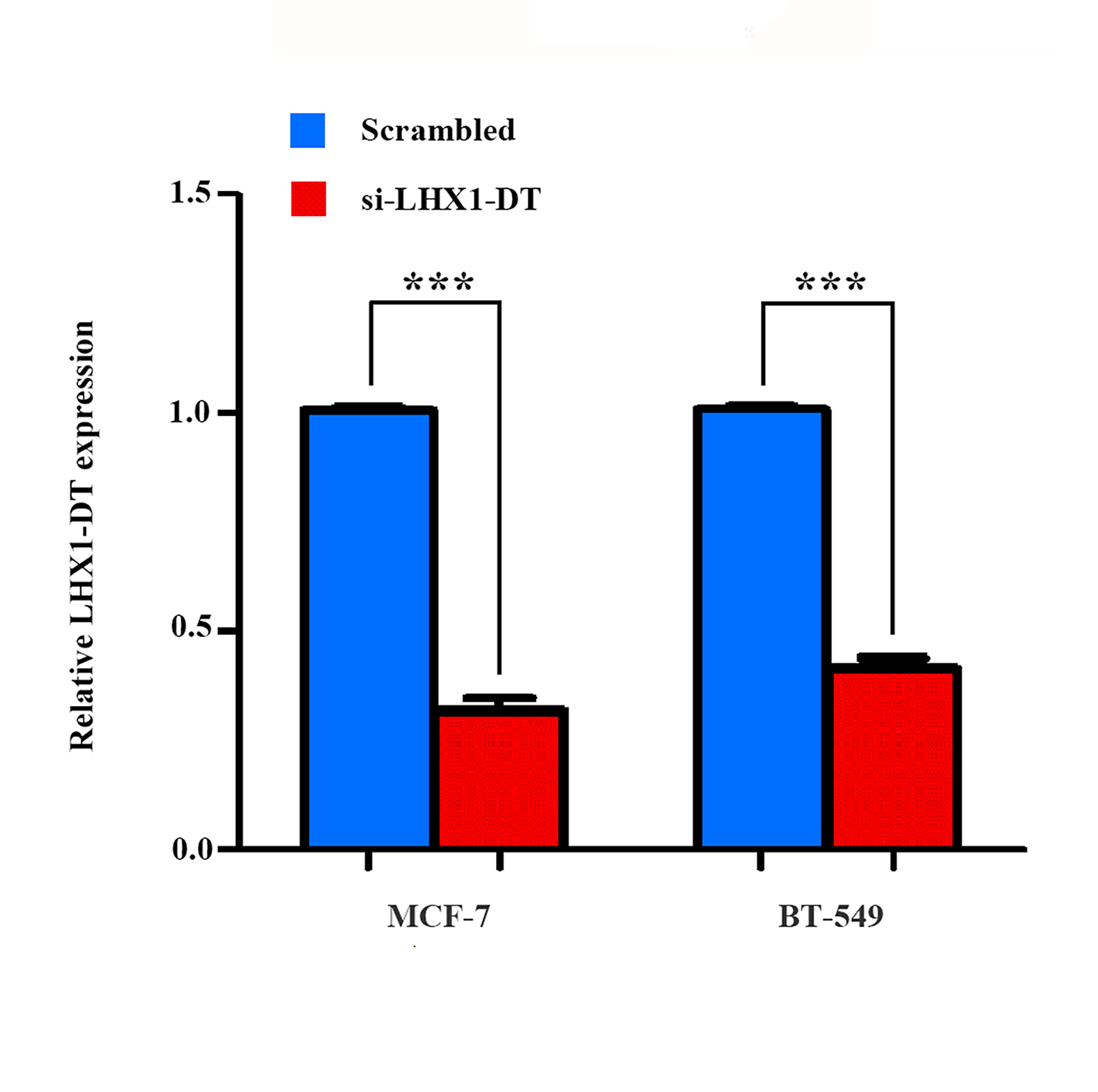

Supplement: Supplementary Figure 3 — The efficiency knockdown of the expression levels of LHX1-DT. qRT-PCR detection of LHX1-DT expression in MCF-7 and BT-549 cells transfected with scrambled or si-LHX1-DT. Three independent experiments, Student’s test. qRT-PCR, quantitative real-time polymerase chain reaction. [file Image_3.TIF]
