# Supplementary material for: Individualized Prediction of Survival by a 10-Long Non-coding RNA-Based Prognostic Model for Patients With Breast Cancer
Source: Front Oncol. 2020 Oct 19;10:515421. doi: 10.3389/fonc.2020.515421 (PMC7604500; doi:10.3389/fonc.2020.515421)
Supplement: Supplementary Table 1 — Candidate prognostic lncRNAs in the TCGA-BRCA Project. [file Table_1.DOCX]

**Supplementary Table 1.** Candidate prognostic lncRNAs in the TCGA-BRCA Project

| **Gene name** | **Log rank *P* value** | **Cox *P* value** | **HR** | **95% CI** | |
| --- | --- | --- | --- | --- | --- |
| ADARB2-AS1 | 8.34E-06 | 1.50E-05 | 2.330102 | 1.588824 | 3.417227 |
| AC243773.1 | 3.26E-05 | 6.40E-05 | 2.670161 | 1.649719 | 4.321805 |
| AL513123.1 | 5.42E-05 | 8.60E-05 | 2.341141 | 1.531174 | 3.579568 |
| AC122710.2 | 5.67E-05 | 8.30E-05 | 2.211249 | 1.489228 | 3.283326 |
| TRPC7-AS1 | 0.000140 | 0.00019 | 2.127408 | 1.431192 | 3.162305 |
| AL022329.1 | 0.000411 | 0.000538 | 0.494275 | 0.331648 | 0.736646 |
| AC073359.1 | 0.001563 | 0.002021 | 0.456048 | 0.27703 | 0.750751 |
| PCAT7 | 0.001753 | 0.002064 | 1.858893 | 1.253042 | 2.757676 |
| AC006262.3 | 0.002238 | 0.002688 | 1.952228 | 1.261256 | 3.021746 |
| AL133467.1 | 0.002864 | 0.003273 | 0.557801 | 0.378024 | 0.823074 |
| LINC00536 | 0.002951 | 0.003404 | 1.822583 | 1.219621 | 2.72364 |
| LINC01234 | 0.002993 | 0.003394 | 1.796816 | 1.214054 | 2.659311 |
| AC073342.2 | 0.003271 | 0.003692 | 1.754057 | 1.200321 | 2.563243 |
| AL592043.1 | 0.003333 | 0.004126 | 2.178734 | 1.279699 | 3.709372 |
| LINC01574 | 0.004680 | 0.005238 | 1.765831 | 1.184656 | 2.632122 |
| MIR3150BHG | 0.004931 | 0.005513 | 1.727853 | 1.174308 | 2.542329 |
| AL161646.2 | 0.005378 | 0.005987 | 1.736343 | 1.171525 | 2.573473 |
| AC112721.1 | 0.005578 | 0.006033 | 1.725571 | 1.169012 | 2.547106 |
| WDR86-AS1 | 0.005701 | 0.006376 | 0.584152 | 0.396982 | 0.85957 |
| AC245123.1 | 0.005708 | 0.006465 | 0.54864 | 0.356162 | 0.845137 |
| FP325330.3 | 0.006669 | 0.007381 | 1.690816 | 1.151408 | 2.482925 |
| AL445647.1 | 0.006844 | 0.007584 | 1.743229 | 1.159277 | 2.621331 |
| BCAR4 | 0.006934 | 0.007601 | 1.702203 | 1.15183 | 2.515557 |
| AC022874.1 | 0.007016 | 0.007807 | 1.791467 | 1.165889 | 2.752709 |
| AC090502.1 | 0.007045 | 0.007638 | 1.707799 | 1.152562 | 2.530519 |
| LINC01385 | 0.007171 | 0.007846 | 1.677554 | 1.145627 | 2.456459 |
| AC108474.1 | 0.008707 | 0.00952 | 0.5927 | 0.39913 | 0.880149 |
| C6orf99 | 0.009045 | 0.009878 | 1.65846 | 1.129291 | 2.435589 |
| AC007128.1 | 0.012641 | 0.013531 | 1.629348 | 1.105972 | 2.400399 |
| AC105398.1 | 0.013026 | 0.01468 | 0.5239 | 0.311707 | 0.880542 |
| AL157778.1 | 0.014388 | 0.015358 | 1.610493 | 1.095484 | 2.367618 |
| AC122710.3 | 0.016282 | 0.017357 | 1.591998 | 1.085359 | 2.335133 |
| AC116312.1 | 0.016422 | 0.017262 | 0.626023 | 0.42575 | 0.920504 |
| AC099521.1 | 0.016663 | 0.017447 | 1.600352 | 1.086025 | 2.358258 |
| ACTA2-AS1 | 0.017594 | 0.018819 | 0.62493 | 0.422166 | 0.925079 |
| LINC01977 | 0.018237 | 0.019183 | 1.583626 | 1.077867 | 2.326696 |
| AC246793.1 | 0.019084 | 0.02026 | 1.569037 | 1.072671 | 2.295091 |
| AC010595.1 | 0.019684 | 0.02106 | 1.609949 | 1.074243 | 2.412802 |
| CARMN | 0.019770 | 0.020836 | 0.632477 | 0.428851 | 0.932788 |
| AC027306.1 | 0.020113 | 0.021399 | 0.632662 | 0.428354 | 0.934418 |
| AC079414.1 | 0.021441 | 0.022961 | 1.583932 | 1.065588 | 2.35442 |
| LINC01179 | 0.023241 | 0.024368 | 1.612535 | 1.063778 | 2.444373 |
| AC096888.1 | 0.025342 | 0.026345 | 1.542633 | 1.052285 | 2.261476 |
| LINC01456 | 0.026344 | 0.028 | 1.623064 | 1.053701 | 2.500079 |
| HOXC-AS3 | 0.027272 | 0.028551 | 1.533323 | 1.045854 | 2.248 |
| MME-AS1 | 0.027281 | 0.02899 | 0.592084 | 0.369896 | 0.947734 |
| AL138789.1 | 0.028504 | 0.029618 | 1.52804 | 1.042839 | 2.238989 |
| ADAMTS9-AS1 | 0.029231 | 0.030607 | 0.653615 | 0.444542 | 0.961017 |
| AC093515.1 | 0.029599 | 0.030839 | 1.520084 | 1.039387 | 2.223094 |
| LHX1-DT | 0.030791 | 0.032191 | 1.51631 | 1.036016 | 2.219268 |
| AL589765.7 | 0.032148 | 0.033375 | 1.534002 | 1.034276 | 2.275179 |
| AC097713.1 | 0.032216 | 0.0336 | 0.656134 | 0.444822 | 0.967831 |
| AL591686.1 | 0.032622 | 0.033752 | 0.659149 | 0.448612 | 0.968494 |
| AL596442.1 | 0.034178 | 0.035775 | 1.616727 | 1.032439 | 2.531683 |
| WT1-AS | 0.034320 | 0.035413 | 1.50805 | 1.02845 | 2.211305 |
| AC132807.2 | 0.034461 | 0.036107 | 1.52388 | 1.027664 | 2.259699 |
| RUNDC3A-AS1 | 0.034871 | 0.035839 | 1.507352 | 1.027508 | 2.211284 |
| AP003555.3 | 0.035064 | 0.036517 | 0.657756 | 0.444161 | 0.974066 |
| AL356479.1 | 0.035919 | 0.036796 | 0.664126 | 0.452275 | 0.975212 |
| LINC00200 | 0.037711 | 0.039228 | 1.522517 | 1.020977 | 2.270433 |
| AC018467.1 | 0.038707 | 0.042139 | 0.53587 | 0.29359 | 0.978085 |
| AC099566.1 | 0.040884 | 0.044002 | 0.53924 | 0.295645 | 0.983545 |
| AP005264.1 | 0.042062 | 0.043309 | 0.672793 | 0.458076 | 0.988155 |
| LINC02532 | 0.042323 | 0.043736 | 1.485253 | 1.011174 | 2.181599 |
| AJ003147.1 | 0.042864 | 0.044152 | 1.499003 | 1.010652 | 2.223327 |
| AC068228.1 | 0.042912 | 0.044405 | 1.477065 | 1.009804 | 2.160538 |
| AL139023.1 | 0.043173 | 0.04477 | 1.519877 | 1.00982 | 2.287563 |
| LINC02408 | 0.043302 | 0.044724 | 1.477885 | 1.009242 | 2.164141 |
| AC036108.3 | 0.043954 | 0.045183 | 0.675762 | 0.460511 | 0.991624 |
| AC067930.5 | 0.044957 | 0.045962 | 1.475659 | 1.006999 | 2.162434 |
| AC010099.4 | 0.046619 | 0.047632 | 1.474798 | 1.004063 | 2.166226 |

Abbreviations: lncRNA: Long non-coding RNA; TCGA: The Cancer Genome Atlas; HR, hazard ratio; CI, confidence interval.
