# Supplementary material for: Individualized Prediction of Survival by a 10-Long Non-coding RNA-Based Prognostic Model for Patients With Breast Cancer
Source: Front Oncol. 2020 Oct 19;10:515421. doi: 10.3389/fonc.2020.515421 (PMC7604500; doi:10.3389/fonc.2020.515421)
Supplement: Supplementary Table 2 — Enrichment analysis of GO terms for positively co-expressed DEMs correlated with prognostic lncRNAs. [file Table_2.DOCX]

**Supplementary Table 2.** Enrichment analysis of GO terms for positively co-expressed DEMs correlated with prognostic lncRNAs

| **ID** | **Function Description** | ***P* value** | **GeneRatio** | **BgRatio** |
| --- | --- | --- | --- | --- |
| GO:0007059 | chromosome segregation | 1.65E-42 | 48/193 | 312/18493 |
| GO:0140014 | mitotic nuclear division | 1.58E-39 | 44/193 | 277/18493 |
| GO:0000280 | nuclear division | 6.96E-39 | 50/193 | 416/18493 |
| GO:0048285 | organelle fission | 5.26E-38 | 51/193 | 458/18493 |
| GO:0098813 | nuclear chromosome segregation | 8.29E-37 | 41/193 | 258/18493 |
| GO:0000819 | sister chromatid segregation | 1.24E-35 | 36/193 | 185/18493 |
| GO:0000070 | mitotic sister chromatid segregation | 2.89E-34 | 33/193 | 153/18493 |
| GO:0007088 | regulation of mitotic nuclear division | 7.28E-24 | 27/193 | 177/18493 |
| GO:0051783 | regulation of nuclear division | 1.35E-23 | 28/193 | 201/18493 |
| GO:0000075 | cell cycle checkpoint | 1.02E-22 | 28/193 | 216/18493 |
| GO:1901990 | regulation of mitotic cell cycle phase transition | 2.66E-22 | 34/193 | 375/18493 |
| GO:0007051 | spindle organization | 4.41E-22 | 25/193 | 165/18493 |
| GO:1902850 | microtubule cytoskeleton organization involved in mitosis | 4.53E-22 | 23/193 | 129/18493 |
| GO:1901987 | regulation of cell cycle phase transition | 5.22E-22 | 35/193 | 412/18493 |
| GO:0051983 | regulation of chromosome segregation | 1.57E-21 | 21/193 | 103/18493 |
| GO:0007052 | mitotic spindle organization | 1.14E-19 | 20/193 | 108/18493 |
| GO:0007093 | mitotic cell cycle checkpoint | 1.75E-19 | 23/193 | 166/18493 |
| GO:0051304 | chromosome separation | 1.53E-18 | 18/193 | 89/18493 |
| GO:0030071 | regulation of mitotic metaphase/anaphase transition | 2.51E-18 | 15/193 | 51/18493 |
| GO:1902099 | regulation of metaphase/anaphase transition of cell cycle | 4.84E-18 | 15/193 | 53/18493 |
| GO:0007091 | metaphase/anaphase transition of mitotic cell cycle | 6.63E-18 | 15/193 | 54/18493 |
| GO:0044784 | metaphase/anaphase transition of cell cycle | 1.22E-17 | 15/193 | 56/18493 |
| GO:0010965 | regulation of mitotic sister chromatid separation | 1.65E-17 | 15/193 | 57/18493 |
| GO:0051306 | mitotic sister chromatid separation | 3.86E-17 | 15/193 | 60/18493 |
| GO:1903046 | meiotic cell cycle process | 4.53E-17 | 22/193 | 188/18493 |
| GO:1905818 | regulation of chromosome separation | 6.63E-17 | 15/193 | 62/18493 |
| GO:0010948 | negative regulation of cell cycle process | 1.36E-16 | 26/193 | 304/18493 |
| GO:0051321 | meiotic cell cycle | 1.43E-16 | 24/193 | 249/18493 |
| GO:0033045 | regulation of sister chromatid segregation | 1.59E-16 | 16/193 | 80/18493 |
| GO:0033047 | regulation of mitotic sister chromatid segregation | 3.00E-16 | 15/193 | 68/18493 |
| GO:1901988 | negative regulation of cell cycle phase transition | 5.39E-16 | 22/193 | 211/18493 |
| GO:0045787 | positive regulation of cell cycle | 7.87E-16 | 28/193 | 390/18493 |
| GO:1901991 | negative regulation of mitotic cell cycle phase transition | 8.73E-16 | 21/193 | 191/18493 |
| GO:0044839 | cell cycle G2/M phase transition | 1.08E-15 | 22/193 | 218/18493 |
| GO:0000086 | G2/M transition of mitotic cell cycle | 2.02E-15 | 21/193 | 199/18493 |
| GO:0045930 | negative regulation of mitotic cell cycle | 2.25E-15 | 24/193 | 281/18493 |
| GO:0090068 | positive regulation of cell cycle process | 3.34E-15 | 24/193 | 286/18493 |
| GO:0140013 | meiotic nuclear division | 1.80E-14 | 19/193 | 171/18493 |
| GO:0033046 | negative regulation of sister chromatid segregation | 1.86E-14 | 12/193 | 44/18493 |
| GO:0051310 | metaphase plate congression | 1.90E-14 | 13/193 | 57/18493 |
| GO:0051985 | negative regulation of chromosome segregation | 2.52E-14 | 12/193 | 45/18493 |
| GO:0007094 | mitotic spindle assembly checkpoint | 2.79E-14 | 11/193 | 34/18493 |
| GO:0031577 | spindle checkpoint | 2.79E-14 | 11/193 | 34/18493 |
| GO:0071173 | spindle assembly checkpoint | 2.79E-14 | 11/193 | 34/18493 |
| GO:0071174 | mitotic spindle checkpoint | 2.79E-14 | 11/193 | 34/18493 |
| GO:0051303 | establishment of chromosome localization | 2.98E-14 | 14/193 | 74/18493 |
| GO:0050000 | chromosome localization | 3.63E-14 | 14/193 | 75/18493 |
| GO:0045841 | negative regulation of mitotic metaphase/anaphase transition | 5.75E-14 | 11/193 | 36/18493 |
| GO:1902100 | negative regulation of metaphase/anaphase transition of cell cycle | 8.11E-14 | 11/193 | 37/18493 |
| GO:2000816 | negative regulation of mitotic sister chromatid separation | 1.56E-13 | 11/193 | 39/18493 |
| GO:1902749 | regulation of cell cycle G2/M phase transition | 2.10E-13 | 18/193 | 170/18493 |
| GO:1905819 | negative regulation of chromosome separation | 2.13E-13 | 11/193 | 40/18493 |
| GO:0033048 | negative regulation of mitotic sister chromatid segregation | 3.88E-13 | 11/193 | 42/18493 |
| GO:0045132 | meiotic chromosome segregation | 4.35E-13 | 14/193 | 89/18493 |
| GO:0010389 | regulation of G2/M transition of mitotic cell cycle | 4.54E-13 | 17/193 | 153/18493 |
| GO:0008608 | attachment of spindle microtubules to kinetochore | 6.41E-13 | 10/193 | 32/18493 |
| GO:0051784 | negative regulation of nuclear division | 1.07E-12 | 12/193 | 60/18493 |
| GO:0031145 | anaphase-promoting complex-dependent catabolic process | 3.31E-12 | 10/193 | 37/18493 |
| GO:0051225 | spindle assembly | 4.53E-12 | 14/193 | 105/18493 |
| GO:0045839 | negative regulation of mitotic nuclear division | 5.00E-12 | 11/193 | 52/18493 |
| GO:0031570 | DNA integrity checkpoint | 9.54E-12 | 16/193 | 158/18493 |
| GO:0000910 | cytokinesis | 1.54E-11 | 16/193 | 163/18493 |
| GO:0007080 | mitotic metaphase plate congression | 2.21E-11 | 10/193 | 44/18493 |
| GO:1901989 | positive regulation of cell cycle phase transition | 3.73E-11 | 13/193 | 100/18493 |
| GO:0033044 | regulation of chromosome organization | 3.97E-11 | 21/193 | 330/18493 |
| GO:0006260 | DNA replication | 5.61E-11 | 19/193 | 268/18493 |
| GO:0045931 | positive regulation of mitotic cell cycle | 1.00E-10 | 15/193 | 157/18493 |
| GO:0044843 | cell cycle G1/S phase transition | 1.35E-10 | 19/193 | 282/18493 |
| GO:2001251 | negative regulation of chromosome organization | 3.41E-10 | 14/193 | 144/18493 |
| GO:0090307 | mitotic spindle assembly | 5.82E-10 | 10/193 | 60/18493 |
| GO:0034502 | protein localization to chromosome | 7.72E-10 | 11/193 | 81/18493 |
| GO:0044774 | mitotic DNA integrity checkpoint | 1.24E-09 | 12/193 | 107/18493 |
| GO:1901992 | positive regulation of mitotic cell cycle phase transition | 1.31E-09 | 11/193 | 85/18493 |
| GO:0071459 | protein localization to chromosome, centromeric region | 1.81E-09 | 7/193 | 22/18493 |
| GO:0000082 | G1/S transition of mitotic cell cycle | 2.48E-09 | 17/193 | 263/18493 |
| GO:0061982 | meiosis I cell cycle process | 3.85E-09 | 12/193 | 118/18493 |
| GO:0007062 | sister chromatid cohesion | 6.05E-09 | 9/193 | 56/18493 |
| GO:0071103 | DNA conformation change | 7.46E-09 | 17/193 | 283/18493 |
| GO:1902750 | negative regulation of cell cycle G2/M phase transition | 1.14E-08 | 9/193 | 60/18493 |
| GO:0051302 | regulation of cell division | 1.55E-08 | 13/193 | 162/18493 |
| GO:0032465 | regulation of cytokinesis | 1.72E-08 | 10/193 | 84/18493 |
| GO:0000079 | regulation of cyclin-dependent protein serine/threonine kinase activity | 2.42E-08 | 10/193 | 87/18493 |
| GO:0006270 | DNA replication initiation | 3.26E-08 | 7/193 | 32/18493 |
| GO:1904029 | regulation of cyclin-dependent protein kinase activity | 3.75E-08 | 10/193 | 91/18493 |
| GO:0051656 | establishment of organelle localization | 4.84E-08 | 21/193 | 491/18493 |
| GO:0006261 | DNA-dependent DNA replication | 5.02E-08 | 12/193 | 148/18493 |
| GO:0061640 | cytoskeleton-dependent cytokinesis | 7.67E-08 | 10/193 | 98/18493 |
| GO:0000281 | mitotic cytokinesis | 2.53E-07 | 9/193 | 85/18493 |
| GO:0007127 | meiosis I | 2.97E-07 | 10/193 | 113/18493 |
| GO:0070192 | chromosome organization involved in meiotic cell cycle | 3.27E-07 | 8/193 | 64/18493 |
| GO:0000077 | DNA damage checkpoint | 3.87E-07 | 11/193 | 146/18493 |
| GO:0030261 | chromosome condensation | 3.92E-07 | 7/193 | 45/18493 |
| GO:0000076 | DNA replication checkpoint | 4.68E-07 | 5/193 | 16/18493 |
| GO:0007143 | female meiotic nuclear division | 5.76E-07 | 6/193 | 30/18493 |
| GO:0010972 | negative regulation of G2/M transition of mitotic cell cycle | 6.20E-07 | 7/193 | 48/18493 |
| GO:0051445 | regulation of meiotic cell cycle | 6.20E-07 | 7/193 | 48/18493 |
| GO:0034501 | protein localization to kinetochore | 6.57E-07 | 5/193 | 17/18493 |
| GO:0072401 | signal transduction involved in DNA integrity checkpoint | 9.16E-07 | 8/193 | 73/18493 |
| GO:0072422 | signal transduction involved in DNA damage checkpoint | 9.16E-07 | 8/193 | 73/18493 |
| GO:0072395 | signal transduction involved in cell cycle checkpoint | 1.02E-06 | 8/193 | 74/18493 |
| GO:0006302 | double-strand break repair | 1.23E-06 | 13/193 | 236/18493 |
| GO:0034508 | centromere complex assembly | 1.82E-06 | 7/193 | 56/18493 |
| GO:0006323 | DNA packaging | 2.08E-06 | 12/193 | 209/18493 |
| GO:0051383 | kinetochore organization | 2.09E-06 | 5/193 | 21/18493 |
| GO:0033314 | mitotic DNA replication checkpoint | 2.30E-06 | 4/193 | 10/18493 |
| GO:0010639 | negative regulation of organelle organization | 2.90E-06 | 16/193 | 384/18493 |
| GO:1902806 | regulation of cell cycle G1/S phase transition | 3.99E-06 | 11/193 | 185/18493 |
| GO:0051307 | meiotic chromosome separation | 5.27E-06 | 5/193 | 25/18493 |
| GO:0000022 | mitotic spindle elongation | 5.33E-06 | 4/193 | 12/18493 |
| GO:0007077 | mitotic nuclear envelope disassembly | 5.33E-06 | 4/193 | 12/18493 |
| GO:0051231 | spindle elongation | 7.64E-06 | 4/193 | 13/18493 |
| GO:0051255 | spindle midzone assembly | 7.64E-06 | 4/193 | 13/18493 |
| GO:0051315 | attachment of mitotic spindle microtubules to kinetochore | 7.64E-06 | 4/193 | 13/18493 |
| GO:0044773 | mitotic DNA damage checkpoint | 8.59E-06 | 8/193 | 98/18493 |
| GO:0042770 | signal transduction in response to DNA damage | 9.05E-06 | 9/193 | 130/18493 |
| GO:0010971 | positive regulation of G2/M transition of mitotic cell cycle | 9.51E-06 | 5/193 | 28/18493 |
| GO:2000045 | regulation of G1/S transition of mitotic cell cycle | 9.86E-06 | 10/193 | 166/18493 |
| GO:0007292 | female gamete generation | 1.16E-05 | 9/193 | 134/18493 |
| GO:0045840 | positive regulation of mitotic nuclear division | 1.46E-05 | 6/193 | 51/18493 |
| GO:0070507 | regulation of microtubule cytoskeleton organization | 1.56E-05 | 10/193 | 175/18493 |
| GO:0044818 | mitotic G2/M transition checkpoint | 1.60E-05 | 5/193 | 31/18493 |
| GO:1902751 | positive regulation of cell cycle G2/M phase transition | 1.60E-05 | 5/193 | 31/18493 |
| GO:0044380 | protein localization to cytoskeleton | 1.83E-05 | 6/193 | 53/18493 |
| GO:2000241 | regulation of reproductive process | 1.84E-05 | 9/193 | 142/18493 |
| GO:0007076 | mitotic chromosome condensation | 1.90E-05 | 4/193 | 16/18493 |
| GO:0030397 | membrane disassembly | 1.90E-05 | 4/193 | 16/18493 |
| GO:0051081 | nuclear envelope disassembly | 1.90E-05 | 4/193 | 16/18493 |
| GO:0022412 | cellular process involved in reproduction in multicellular organism | 1.94E-05 | 14/193 | 350/18493 |
| GO:0016321 | female meiosis chromosome segregation | 2.19E-05 | 3/193 | 6/18493 |
| GO:0051782 | negative regulation of cell division | 2.46E-05 | 4/193 | 17/18493 |
| GO:0006977 | DNA damage response, signal transduction by p53 class mediator resulting in cell cycle arrest | 2.52E-05 | 6/193 | 56/18493 |
| GO:0045143 | homologous chromosome segregation | 2.52E-05 | 6/193 | 56/18493 |
| GO:0007018 | microtubule-based movement | 2.69E-05 | 12/193 | 269/18493 |
| GO:0072431 | signal transduction involved in mitotic G1 DNA damage checkpoint | 2.79E-05 | 6/193 | 57/18493 |
| GO:1902400 | intracellular signal transduction involved in G1 DNA damage checkpoint | 2.79E-05 | 6/193 | 57/18493 |
| GO:0006890 | retrograde vesicle-mediated transport, Golgi to ER | 3.24E-05 | 7/193 | 86/18493 |
| GO:0072413 | signal transduction involved in mitotic cell cycle checkpoint | 3.40E-05 | 6/193 | 59/18493 |
| GO:1902402 | signal transduction involved in mitotic DNA damage checkpoint | 3.40E-05 | 6/193 | 59/18493 |
| GO:1902403 | signal transduction involved in mitotic DNA integrity checkpoint | 3.40E-05 | 6/193 | 59/18493 |
| GO:0007098 | centrosome cycle | 3.55E-05 | 8/193 | 119/18493 |
| GO:0032914 | positive regulation of transforming growth factor beta1 production | 3.80E-05 | 3/193 | 7/18493 |
| GO:0033313 | meiotic cell cycle checkpoint | 3.80E-05 | 3/193 | 7/18493 |
| GO:0016572 | histone phosphorylation | 3.91E-05 | 5/193 | 37/18493 |
| GO:0032467 | positive regulation of cytokinesis | 3.91E-05 | 5/193 | 37/18493 |
| GO:0006310 | DNA recombination | 4.26E-05 | 12/193 | 282/18493 |
| GO:0032506 | cytokinetic process | 4.46E-05 | 5/193 | 38/18493 |
| GO:0031571 | mitotic G1 DNA damage checkpoint | 4.95E-05 | 6/193 | 63/18493 |
| GO:0044819 | mitotic G1/S transition checkpoint | 4.95E-05 | 6/193 | 63/18493 |
| GO:0044783 | G1 DNA damage checkpoint | 5.42E-05 | 6/193 | 64/18493 |
| GO:0051785 | positive regulation of nuclear division | 5.42E-05 | 6/193 | 64/18493 |
| GO:0032886 | regulation of microtubule-based process | 5.80E-05 | 10/193 | 204/18493 |
| GO:1904666 | regulation of ubiquitin protein ligase activity | 5.99E-05 | 4/193 | 21/18493 |
| GO:0044771 | meiotic cell cycle phase transition | 6.03E-05 | 3/193 | 8/18493 |
| GO:0045144 | meiotic sister chromatid segregation | 6.03E-05 | 3/193 | 8/18493 |
| GO:0051177 | meiotic sister chromatid cohesion | 6.03E-05 | 3/193 | 8/18493 |
| GO:1901993 | regulation of meiotic cell cycle phase transition | 6.03E-05 | 3/193 | 8/18493 |
| GO:0071900 | regulation of protein serine/threonine kinase activity | 6.74E-05 | 16/193 | 496/18493 |
| GO:0051653 | spindle localization | 7.31E-05 | 5/193 | 42/18493 |
| GO:0019886 | antigen processing and presentation of exogenous peptide antigen via MHC class II | 7.51E-05 | 7/193 | 98/18493 |
| GO:0031023 | microtubule organizing center organization | 7.84E-05 | 8/193 | 133/18493 |
| GO:1901978 | positive regulation of cell cycle checkpoint | 8.98E-05 | 3/193 | 9/18493 |
| GO:0002495 | antigen processing and presentation of peptide antigen via MHC class II | 9.09E-05 | 7/193 | 101/18493 |
| GO:0034080 | CENP-A containing nucleosome assembly | 9.17E-05 | 5/193 | 44/18493 |
| GO:0061641 | CENP-A containing chromatin organization | 9.17E-05 | 5/193 | 44/18493 |
| GO:0019882 | antigen processing and presentation | 9.47E-05 | 9/193 | 175/18493 |
| GO:0002504 | antigen processing and presentation of peptide or polysaccharide antigen via MHC class II | 9.68E-05 | 7/193 | 102/18493 |
| GO:0072331 | signal transduction by p53 class mediator | 1.01E-04 | 10/193 | 218/18493 |
| GO:0048002 | antigen processing and presentation of peptide antigen | 1.07E-04 | 8/193 | 139/18493 |
| GO:0030330 | DNA damage response, signal transduction by p53 class mediator | 1.09E-04 | 7/193 | 104/18493 |
| GO:0051052 | regulation of DNA metabolic process | 1.13E-04 | 14/193 | 412/18493 |
| GO:0031109 | microtubule polymerization or depolymerization | 1.23E-04 | 7/193 | 106/18493 |
| GO:0007135 | meiosis II | 1.27E-04 | 3/193 | 10/18493 |
| GO:0032908 | regulation of transforming growth factor beta1 production | 1.27E-04 | 3/193 | 10/18493 |
| GO:0051256 | mitotic spindle midzone assembly | 1.27E-04 | 3/193 | 10/18493 |
| GO:0061983 | meiosis II cell cycle process | 1.27E-04 | 3/193 | 10/18493 |
| GO:0071156 | regulation of cell cycle arrest | 1.31E-04 | 7/193 | 107/18493 |
| GO:0031055 | chromatin remodeling at centromere | 1.40E-04 | 5/193 | 48/18493 |
| GO:0072698 | protein localization to microtubule cytoskeleton | 1.40E-04 | 5/193 | 48/18493 |
| GO:0032392 | DNA geometric change | 1.43E-04 | 6/193 | 76/18493 |
| GO:0071168 | protein localization to chromatin | 1.44E-04 | 4/193 | 26/18493 |
| GO:0040001 | establishment of mitotic spindle localization | 1.67E-04 | 4/193 | 27/18493 |
| GO:0032905 | transforming growth factor beta1 production | 1.74E-04 | 3/193 | 11/18493 |
| GO:0035404 | histone-serine phosphorylation | 1.74E-04 | 3/193 | 11/18493 |
| GO:0045835 | negative regulation of meiotic nuclear division | 1.74E-04 | 3/193 | 11/18493 |
| GO:1902969 | mitotic DNA replication | 1.74E-04 | 3/193 | 11/18493 |
| GO:0045620 | negative regulation of lymphocyte differentiation | 1.87E-04 | 5/193 | 51/18493 |
| GO:0051984 | positive regulation of chromosome segregation | 1.94E-04 | 4/193 | 28/18493 |
| GO:2000134 | negative regulation of G1/S transition of mitotic cell cycle | 1.94E-04 | 7/193 | 114/18493 |
| GO:0071158 | positive regulation of cell cycle arrest | 2.17E-04 | 6/193 | 82/18493 |
| GO:0000083 | regulation of transcription involved in G1/S transition of mitotic cell cycle | 2.23E-04 | 4/193 | 29/18493 |
| GO:0007131 | reciprocal meiotic recombination | 2.25E-04 | 5/193 | 53/18493 |
| GO:0035825 | homologous recombination | 2.25E-04 | 5/193 | 53/18493 |
| GO:0007050 | cell cycle arrest | 2.28E-04 | 10/193 | 241/18493 |
| GO:0051988 | regulation of attachment of spindle microtubules to kinetochore | 2.30E-04 | 3/193 | 12/18493 |
| GO:0055015 | ventricular cardiac muscle cell development | 2.30E-04 | 3/193 | 12/18493 |
| GO:0070601 | centromeric sister chromatid cohesion | 2.30E-04 | 3/193 | 12/18493 |
| GO:1904668 | positive regulation of ubiquitin protein ligase activity | 2.30E-04 | 3/193 | 12/18493 |
| GO:0006336 | DNA replication-independent nucleosome assembly | 2.46E-04 | 5/193 | 54/18493 |
| GO:0034724 | DNA replication-independent nucleosome organization | 2.68E-04 | 5/193 | 55/18493 |
| GO:1902807 | negative regulation of cell cycle G1/S phase transition | 2.80E-04 | 7/193 | 121/18493 |
| GO:0040020 | regulation of meiotic nuclear division | 2.90E-04 | 4/193 | 31/18493 |
| GO:0000212 | meiotic spindle organization | 2.96E-04 | 3/193 | 13/18493 |
| GO:0007100 | mitotic centrosome separation | 2.96E-04 | 3/193 | 13/18493 |
| GO:0070098 | chemokine-mediated signaling pathway | 2.99E-04 | 6/193 | 87/18493 |
| GO:0008315 | G2/MI transition of meiotic cell cycle | 3.23E-04 | 2/193 | 3/18493 |
| GO:0051598 | meiotic recombination checkpoint | 3.23E-04 | 2/193 | 3/18493 |
| GO:0051710 | regulation of cytolysis in other organism | 3.23E-04 | 2/193 | 3/18493 |
| GO:0051714 | positive regulation of cytolysis in other organism | 3.23E-04 | 2/193 | 3/18493 |
| GO:0090360 | platelet-derived growth factor production | 3.23E-04 | 2/193 | 3/18493 |
| GO:0090361 | regulation of platelet-derived growth factor production | 3.23E-04 | 2/193 | 3/18493 |
| GO:0110030 | regulation of G2/MI transition of meiotic cell cycle | 3.23E-04 | 2/193 | 3/18493 |
| GO:0110032 | positive regulation of G2/MI transition of meiotic cell cycle | 3.23E-04 | 2/193 | 3/18493 |
| GO:0031572 | G2 DNA damage checkpoint | 3.29E-04 | 4/193 | 32/18493 |
| GO:0043486 | histone exchange | 3.44E-04 | 5/193 | 58/18493 |
| GO:0002478 | antigen processing and presentation of exogenous peptide antigen | 3.59E-04 | 7/193 | 126/18493 |
| GO:1901976 | regulation of cell cycle checkpoint | 3.71E-04 | 4/193 | 33/18493 |
| GO:0051299 | centrosome separation | 3.74E-04 | 3/193 | 14/18493 |
| GO:0090231 | regulation of spindle checkpoint | 3.74E-04 | 3/193 | 14/18493 |
| GO:0090266 | regulation of mitotic cell cycle spindle assembly checkpoint | 3.74E-04 | 3/193 | 14/18493 |
| GO:1903504 | regulation of mitotic spindle checkpoint | 3.74E-04 | 3/193 | 14/18493 |
| GO:1990868 | response to chemokine | 4.81E-04 | 6/193 | 95/18493 |
| GO:1990869 | cellular response to chemokine | 4.81E-04 | 6/193 | 95/18493 |
| GO:0019884 | antigen processing and presentation of exogenous antigen | 5.20E-04 | 7/193 | 134/18493 |
| GO:0051382 | kinetochore assembly | 5.67E-04 | 3/193 | 16/18493 |
| GO:0051447 | negative regulation of meiotic cell cycle | 5.67E-04 | 3/193 | 16/18493 |
| GO:0051293 | establishment of spindle localization | 5.80E-04 | 4/193 | 37/18493 |
| GO:0032508 | DNA duplex unwinding | 6.27E-04 | 5/193 | 66/18493 |
| GO:0007079 | mitotic chromosome movement towards spindle pole | 6.41E-04 | 2/193 | 4/18493 |
| GO:0032824 | negative regulation of natural killer cell differentiation | 6.41E-04 | 2/193 | 4/18493 |
| GO:0032827 | negative regulation of natural killer cell differentiation involved in immune response | 6.41E-04 | 2/193 | 4/18493 |
| GO:1905448 | positive regulation of mitochondrial ATP synthesis coupled electron transport | 6.41E-04 | 2/193 | 4/18493 |
| GO:0055012 | ventricular cardiac muscle cell differentiation | 6.83E-04 | 3/193 | 17/18493 |
| GO:0071478 | cellular response to radiation | 7.64E-04 | 8/193 | 186/18493 |
| GO:0061351 | neural precursor cell proliferation | 7.66E-04 | 7/193 | 143/18493 |
| GO:0009314 | response to radiation | 7.96E-04 | 13/193 | 443/18493 |
| GO:0007096 | regulation of exit from mitosis | 8.14E-04 | 3/193 | 18/18493 |
| GO:0043161 | proteasome-mediated ubiquitin-dependent protein catabolic process | 8.21E-04 | 12/193 | 389/18493 |
| GO:0006275 | regulation of DNA replication | 9.02E-04 | 6/193 | 107/18493 |
| GO:2000779 | regulation of double-strand break repair | 9.34E-04 | 5/193 | 72/18493 |
| GO:0045737 | positive regulation of cyclin-dependent protein serine/threonine kinase activity | 9.59E-04 | 3/193 | 19/18493 |
| GO:0071636 | positive regulation of transforming growth factor beta production | 9.59E-04 | 3/193 | 19/18493 |
| GO:0002325 | natural killer cell differentiation involved in immune response | 1.06E-03 | 2/193 | 5/18493 |
| GO:0002361 | CD4-positive, CD25-positive, alpha-beta regulatory T cell differentiation | 1.06E-03 | 2/193 | 5/18493 |
| GO:0031536 | positive regulation of exit from mitosis | 1.06E-03 | 2/193 | 5/18493 |
| GO:0032826 | regulation of natural killer cell differentiation involved in immune response | 1.06E-03 | 2/193 | 5/18493 |
| GO:0051754 | meiotic sister chromatid cohesion, centromeric | 1.06E-03 | 2/193 | 5/18493 |
| GO:1901995 | positive regulation of meiotic cell cycle phase transition | 1.06E-03 | 2/193 | 5/18493 |
| GO:0002244 | hematopoietic progenitor cell differentiation | 1.09E-03 | 6/193 | 111/18493 |
| GO:0032653 | regulation of interleukin-10 production | 1.23E-03 | 4/193 | 45/18493 |
| GO:0048245 | eosinophil chemotaxis | 1.30E-03 | 3/193 | 21/18493 |
| GO:0007129 | synapsis | 1.33E-03 | 4/193 | 46/18493 |
| GO:0051209 | release of sequestered calcium ion into cytosol | 1.37E-03 | 6/193 | 116/18493 |
| GO:0065004 | protein-DNA complex assembly | 1.43E-03 | 9/193 | 254/18493 |
| GO:0032613 | interleukin-10 production | 1.44E-03 | 4/193 | 47/18493 |
| GO:0071456 | cellular response to hypoxia | 1.47E-03 | 7/193 | 160/18493 |
| GO:0007063 | regulation of sister chromatid cohesion | 1.49E-03 | 3/193 | 22/18493 |
| GO:0007064 | mitotic sister chromatid cohesion | 1.49E-03 | 3/193 | 22/18493 |
| GO:0051283 | negative regulation of sequestering of calcium ion | 1.50E-03 | 6/193 | 118/18493 |
| GO:0021695 | cerebellar cortex development | 1.56E-03 | 4/193 | 48/18493 |
| GO:0000706 | meiotic DNA double-strand break processing | 1.58E-03 | 2/193 | 6/18493 |
| GO:0000912 | assembly of actomyosin apparatus involved in cytokinesis | 1.58E-03 | 2/193 | 6/18493 |
| GO:0000915 | actomyosin contractile ring assembly | 1.58E-03 | 2/193 | 6/18493 |
| GO:0021631 | optic nerve morphogenesis | 1.58E-03 | 2/193 | 6/18493 |
| GO:0090306 | spindle assembly involved in meiosis | 1.58E-03 | 2/193 | 6/18493 |
| GO:1901857 | positive regulation of cellular respiration | 1.58E-03 | 2/193 | 6/18493 |
| GO:1903862 | positive regulation of oxidative phosphorylation | 1.58E-03 | 2/193 | 6/18493 |
| GO:1905446 | regulation of mitochondrial ATP synthesis coupled electron transport | 1.58E-03 | 2/193 | 6/18493 |
| GO:2000371 | regulation of DNA topoisomerase (ATP-hydrolyzing) activity | 1.58E-03 | 2/193 | 6/18493 |
| GO:2000373 | positive regulation of DNA topoisomerase (ATP-hydrolyzing) activity | 1.58E-03 | 2/193 | 6/18493 |
| GO:2001015 | negative regulation of skeletal muscle cell differentiation | 1.58E-03 | 2/193 | 6/18493 |
| GO:0043044 | ATP-dependent chromatin remodeling | 1.59E-03 | 5/193 | 81/18493 |
| GO:0051282 | regulation of sequestering of calcium ion | 1.63E-03 | 6/193 | 120/18493 |
| GO:0006998 | nuclear envelope organization | 1.69E-03 | 4/193 | 49/18493 |
| GO:0002695 | negative regulation of leukocyte activation | 1.70E-03 | 7/193 | 164/18493 |
| GO:0070193 | synaptonemal complex organization | 1.70E-03 | 3/193 | 23/18493 |
| GO:1904031 | positive regulation of cyclin-dependent protein kinase activity | 1.70E-03 | 3/193 | 23/18493 |
| GO:0006338 | chromatin remodeling | 1.76E-03 | 7/193 | 165/18493 |
| GO:0000724 | double-strand break repair via homologous recombination | 1.85E-03 | 6/193 | 123/18493 |
| GO:0051208 | sequestering of calcium ion | 1.85E-03 | 6/193 | 123/18493 |
| GO:0051781 | positive regulation of cell division | 1.86E-03 | 5/193 | 84/18493 |
| GO:2000177 | regulation of neural precursor cell proliferation | 1.86E-03 | 5/193 | 84/18493 |
| GO:0000725 | recombinational repair | 1.93E-03 | 6/193 | 124/18493 |
| GO:0036297 | interstrand cross-link repair | 1.96E-03 | 4/193 | 51/18493 |
| GO:0048477 | oogenesis | 1.96E-03 | 5/193 | 85/18493 |
| GO:0036294 | cellular response to decreased oxygen levels | 2.08E-03 | 7/193 | 170/18493 |
| GO:2000179 | positive regulation of neural precursor cell proliferation | 2.11E-03 | 4/193 | 52/18493 |
| GO:0001556 | oocyte maturation | 2.17E-03 | 3/193 | 25/18493 |
| GO:0072677 | eosinophil migration | 2.17E-03 | 3/193 | 25/18493 |
| GO:0000270 | peptidoglycan metabolic process | 2.20E-03 | 2/193 | 7/18493 |
| GO:0009253 | peptidoglycan catabolic process | 2.20E-03 | 2/193 | 7/18493 |
| GO:0032466 | negative regulation of cytokinesis | 2.20E-03 | 2/193 | 7/18493 |
| GO:0044837 | actomyosin contractile ring organization | 2.20E-03 | 2/193 | 7/18493 |
| GO:0051305 | chromosome movement towards spindle pole | 2.20E-03 | 2/193 | 7/18493 |
| GO:0060059 | embryonic retina morphogenesis in camera-type eye | 2.20E-03 | 2/193 | 7/18493 |
| GO:0090232 | positive regulation of spindle checkpoint | 2.20E-03 | 2/193 | 7/18493 |
| GO:0090267 | positive regulation of mitotic cell cycle spindle assembly checkpoint | 2.20E-03 | 2/193 | 7/18493 |
| GO:1902808 | positive regulation of cell cycle G1/S phase transition | 2.26E-03 | 4/193 | 53/18493 |
| GO:0002285 | lymphocyte activation involved in immune response | 2.37E-03 | 7/193 | 174/18493 |
| GO:0051438 | regulation of ubiquitin-protein transferase activity | 2.42E-03 | 4/193 | 54/18493 |
| GO:0097553 | calcium ion transmembrane import into cytosol | 2.64E-03 | 6/193 | 132/18493 |
| GO:0046605 | regulation of centrosome cycle | 2.77E-03 | 4/193 | 56/18493 |
| GO:0010498 | proteasomal protein catabolic process | 2.89E-03 | 12/193 | 452/18493 |
| GO:0018105 | peptidyl-serine phosphorylation | 2.91E-03 | 9/193 | 282/18493 |
| GO:0007144 | female meiosis I | 2.91E-03 | 2/193 | 8/18493 |
| GO:0032815 | negative regulation of natural killer cell activation | 2.91E-03 | 2/193 | 8/18493 |
| GO:0046602 | regulation of mitotic centrosome separation | 2.91E-03 | 2/193 | 8/18493 |
| GO:0051250 | negative regulation of lymphocyte activation | 3.18E-03 | 6/193 | 137/18493 |
| GO:0050866 | negative regulation of cell activation | 3.24E-03 | 7/193 | 184/18493 |
| GO:0071356 | cellular response to tumor necrosis factor | 3.33E-03 | 8/193 | 235/18493 |
| GO:0021549 | cerebellum development | 3.34E-03 | 5/193 | 96/18493 |
| GO:0071539 | protein localization to centrosome | 3.35E-03 | 3/193 | 29/18493 |
| GO:0030098 | lymphocyte differentiation | 3.38E-03 | 10/193 | 344/18493 |
| GO:0071824 | protein-DNA complex subunit organization | 3.50E-03 | 9/193 | 290/18493 |
| GO:0071453 | cellular response to oxygen levels | 3.54E-03 | 7/193 | 187/18493 |
| GO:1902106 | negative regulation of leukocyte differentiation | 3.65E-03 | 5/193 | 98/18493 |
| GO:0010458 | exit from mitosis | 3.69E-03 | 3/193 | 30/18493 |
| GO:0021696 | cerebellar cortex morphogenesis | 3.69E-03 | 3/193 | 30/18493 |
| GO:0032733 | positive regulation of interleukin-10 production | 3.69E-03 | 3/193 | 30/18493 |
| GO:0032814 | regulation of natural killer cell activation | 3.69E-03 | 3/193 | 30/18493 |
| GO:1905508 | protein localization to microtubule organizing center | 3.69E-03 | 3/193 | 30/18493 |
| GO:0010911 | regulation of isomerase activity | 3.72E-03 | 2/193 | 9/18493 |
| GO:0010912 | positive regulation of isomerase activity | 3.72E-03 | 2/193 | 9/18493 |
| GO:0021683 | cerebellar granular layer morphogenesis | 3.72E-03 | 2/193 | 9/18493 |
| GO:0045842 | positive regulation of mitotic metaphase/anaphase transition | 3.72E-03 | 2/193 | 9/18493 |
| GO:0051715 | cytolysis in other organism | 3.72E-03 | 2/193 | 9/18493 |
| GO:0070314 | G1 to G0 transition | 3.72E-03 | 2/193 | 9/18493 |
| GO:1901970 | positive regulation of mitotic sister chromatid separation | 3.72E-03 | 2/193 | 9/18493 |
| GO:0044786 | cell cycle DNA replication | 4.00E-03 | 4/193 | 62/18493 |
| GO:0002323 | natural killer cell activation involved in immune response | 4.06E-03 | 3/193 | 31/18493 |
| GO:0032689 | negative regulation of interferon-gamma production | 4.06E-03 | 3/193 | 31/18493 |
| GO:0030593 | neutrophil chemotaxis | 4.15E-03 | 5/193 | 101/18493 |
| GO:0048247 | lymphocyte chemotaxis | 4.24E-03 | 4/193 | 63/18493 |
| GO:0010212 | response to ionizing radiation | 4.34E-03 | 6/193 | 146/18493 |
| GO:0018209 | peptidyl-serine modification | 4.46E-03 | 9/193 | 301/18493 |
| GO:0002548 | monocyte chemotaxis | 4.49E-03 | 4/193 | 64/18493 |
| GO:1902101 | positive regulation of metaphase/anaphase transition of cell cycle | 4.61E-03 | 2/193 | 10/18493 |
| GO:0060402 | calcium ion transport into cytosol | 4.79E-03 | 6/193 | 149/18493 |
| GO:0051443 | positive regulation of ubiquitin-protein transferase activity | 4.85E-03 | 3/193 | 33/18493 |
| GO:0071312 | cellular response to alkaloid | 4.85E-03 | 3/193 | 33/18493 |
| GO:0022037 | metencephalon development | 4.89E-03 | 5/193 | 105/18493 |
| GO:0071392 | cellular response to estradiol stimulus | 5.28E-03 | 3/193 | 34/18493 |
| GO:0071634 | regulation of transforming growth factor beta production | 5.28E-03 | 3/193 | 34/18493 |
| GO:0008017 | microtubule binding | 1.52E-12 | 20/190 | 240/17632 |
| GO:0015631 | tubulin binding | 5.27E-11 | 21/190 | 325/17632 |
| GO:0003777 | microtubule motor activity | 1.59E-09 | 11/190 | 84/17632 |
| GO:0003774 | motor activity | 2.77E-07 | 11/190 | 137/17632 |
| GO:0003688 | DNA replication origin binding | 7.68E-07 | 5/190 | 17/17632 |
| GO:0140097 | catalytic activity, acting on DNA | 7.96E-07 | 12/190 | 185/17632 |
| GO:0016887 | ATPase activity | 6.79E-06 | 17/190 | 445/17632 |
| GO:0035173 | histone kinase activity | 2.79E-05 | 4/190 | 17/17632 |
| GO:0097472 | cyclin-dependent protein kinase activity | 4.54E-05 | 5/190 | 37/17632 |
| GO:0010997 | anaphase-promoting complex binding | 6.63E-05 | 3/190 | 8/17632 |
| GO:0008574 | ATP-dependent microtubule motor activity, plus-end-directed | 1.62E-04 | 4/190 | 26/17632 |
| GO:0008009 | chemokine activity | 1.79E-04 | 5/190 | 49/17632 |
| GO:0004693 | cyclin-dependent protein serine/threonine kinase activity | 5.88E-04 | 4/190 | 36/17632 |
| GO:0008745 | N-acetylmuramoyl-L-alanine amidase activity | 6.83E-04 | 2/190 | 4/17632 |
| GO:0042379 | chemokine receptor binding | 7.23E-04 | 5/190 | 66/17632 |
| GO:0019894 | kinesin binding | 9.69E-04 | 4/190 | 41/17632 |
| GO:0016019 | peptidoglycan receptor activity | 1.13E-03 | 2/190 | 5/17632 |
| GO:0048020 | CCR chemokine receptor binding | 1.16E-03 | 4/190 | 43/17632 |
| GO:1990939 | ATP-dependent microtubule motor activity | 1.27E-03 | 4/190 | 44/17632 |
| GO:0004674 | protein serine/threonine kinase activity | 1.35E-03 | 13/190 | 455/17632 |
| GO:0035174 | histone serine kinase activity | 1.68E-03 | 2/190 | 6/17632 |
| GO:0043515 | kinetochore binding | 1.68E-03 | 2/190 | 6/17632 |
| GO:0016538 | cyclin-dependent protein serine/threonine kinase regulator activity | 1.76E-03 | 4/190 | 48/17632 |
| GO:0005819 | spindle | 1.09E-31 | 40/196 | 333/19659 |
| GO:0000775 | chromosome, centromeric region | 7.00E-30 | 32/196 | 194/19659 |
| GO:0000793 | condensed chromosome | 5.14E-28 | 32/196 | 221/19659 |
| GO:0000779 | condensed chromosome, centromeric region | 5.63E-28 | 26/196 | 117/19659 |
| GO:0000776 | kinetochore | 2.03E-26 | 26/196 | 133/19659 |
| GO:0098687 | chromosomal region | 3.59E-25 | 35/196 | 346/19659 |
| GO:0000777 | condensed chromosome kinetochore | 8.52E-25 | 23/196 | 104/19659 |
| GO:0000922 | spindle pole | 7.03E-17 | 20/196 | 154/19659 |
| GO:0005876 | spindle microtubule | 1.37E-16 | 14/196 | 54/19659 |
| GO:0072686 | mitotic spindle | 1.81E-16 | 17/196 | 101/19659 |
| GO:0030496 | midbody | 6.32E-16 | 20/196 | 172/19659 |
| GO:0000940 | condensed chromosome outer kinetochore | 4.05E-14 | 8/196 | 12/19659 |
| GO:0005874 | microtubule | 7.71E-14 | 26/196 | 414/19659 |
| GO:0051233 | spindle midzone | 8.21E-13 | 10/196 | 34/19659 |
| GO:0000780 | condensed nuclear chromosome, centromeric region | 9.30E-13 | 9/196 | 24/19659 |
| GO:0005871 | kinesin complex | 4.81E-12 | 11/196 | 54/19659 |
| GO:0005875 | microtubule associated complex | 2.08E-11 | 15/196 | 147/19659 |
| GO:0000794 | condensed nuclear chromosome | 2.15E-11 | 13/196 | 100/19659 |
| GO:0000778 | condensed nuclear chromosome kinetochore | 2.85E-11 | 7/196 | 14/19659 |
| GO:0000942 | condensed nuclear chromosome outer kinetochore | 9.58E-09 | 4/196 | 4/19659 |
| GO:0032133 | chromosome passenger complex | 4.75E-08 | 4/196 | 5/19659 |
| GO:0031262 | Ndc80 complex | 3.88E-06 | 3/196 | 4/19659 |
| GO:1990023 | mitotic spindle midzone | 6.39E-06 | 4/196 | 13/19659 |
| GO:0010369 | chromocenter | 8.87E-06 | 4/196 | 14/19659 |
| GO:0045120 | pronucleus | 2.06E-05 | 4/196 | 17/19659 |
| GO:0045171 | intercellular bridge | 2.16E-05 | 6/196 | 57/19659 |
| GO:0000307 | cyclin-dependent protein kinase holoenzyme complex | 5.24E-05 | 5/196 | 41/19659 |
| GO:0005814 | centriole | 5.73E-05 | 8/196 | 133/19659 |
| GO:0044450 | microtubule organizing center part | 7.99E-05 | 9/196 | 179/19659 |
| GO:0097125 | cyclin B1-CDK1 complex | 9.89E-05 | 2/196 | 2/19659 |
| GO:0097431 | mitotic spindle pole | 1.41E-04 | 4/196 | 27/19659 |
| GO:1990752 | microtubule end | 1.63E-04 | 4/196 | 28/19659 |
| GO:0031616 | spindle pole centrosome | 3.28E-04 | 3/196 | 14/19659 |
| GO:0005720 | nuclear heterochromatin | 3.52E-04 | 4/196 | 34/19659 |
| GO:0031261 | DNA replication preinitiation complex | 9.70E-04 | 2/196 | 5/19659 |
| GO:0061673 | mitotic spindle astral microtubule | 9.70E-04 | 2/196 | 5/19659 |
| GO:0035371 | microtubule plus-end | 9.82E-04 | 3/196 | 20/19659 |
| GO:0005680 | anaphase-promoting complex | 1.14E-03 | 3/196 | 21/19659 |
| GO:1902554 | serine/threonine protein kinase complex | 1.79E-03 | 5/196 | 87/19659 |
| GO:0005828 | kinetochore microtubule | 2.01E-03 | 2/196 | 7/19659 |
| GO:0090543 | Flemming body | 2.66E-03 | 3/196 | 28/19659 |
| GO:0000235 | astral microtubule | 2.66E-03 | 2/196 | 8/19659 |
| GO:0000796 | condensin complex | 2.66E-03 | 2/196 | 8/19659 |
| GO:0005818 | aster | 2.66E-03 | 2/196 | 8/19659 |
| GO:0031298 | replication fork protection complex | 2.66E-03 | 2/196 | 8/19659 |
| GO:0043203 | axon hillock | 2.66E-03 | 2/196 | 8/19659 |
| GO:0000808 | origin recognition complex | 3.40E-03 | 2/196 | 9/19659 |
| GO:0005664 | nuclear origin of replication recognition complex | 3.40E-03 | 2/196 | 9/19659 |
| GO:0031618 | nuclear pericentric heterochromatin | 3.40E-03 | 2/196 | 9/19659 |
| GO:0070938 | contractile ring | 4.22E-03 | 2/196 | 10/19659 |
| GO:0072687 | meiotic spindle | 4.22E-03 | 2/196 | 10/19659 |
| GO:0005881 | cytoplasmic microtubule | 4.27E-03 | 4/196 | 66/19659 |
| GO:1902911 | protein kinase complex | 4.38E-03 | 5/196 | 107/19659 |
| GO:0000795 | synaptonemal complex | 6.85E-03 | 3/196 | 39/19659 |
| GO:0099086 | synaptonemal structure | 6.85E-03 | 3/196 | 39/19659 |
| GO:0000792 | heterochromatin | 7.03E-03 | 4/196 | 76/19659 |
| GO:0000152 | nuclear ubiquitin ligase complex | 8.99E-03 | 3/196 | 43/19659 |

Abbreviations: GO, gene ontology; DEMs, differently expressed mRNAs; lncRNA: Long non-coding RNA.

Notes:

BGRatio = M/N, M = size of the geneset, N= size of all of the unique genes in the collection of genesets.

GeneRatio = k/n, k = size of the overlap of “a vector of gene id” which input with the specific geneset, n = size of the overlap of “a vector of gene id” which input with all the members of the collection of geneset.
