# Supplementary material for: Individualized Prediction of Survival by a 10-Long Non-coding RNA-Based Prognostic Model for Patients With Breast Cancer
Source: Front Oncol. 2020 Oct 19;10:515421. doi: 10.3389/fonc.2020.515421 (PMC7604500; doi:10.3389/fonc.2020.515421)
Supplement: Supplementary Table 3 — The summary of 10 prognostic lncRNAs. [file Table_3.DOCX]

**Supplementary Table 3.** The summary of 10 prognostic lncRNAs

| **Gene name** | **log_2_FoldChange** | **Down/up-regulated** | ***P* value** |
| --- | --- | --- | --- |
| LHX1-DT | 5.526 | Up | 8.77E-28 |
| AC079414.1 | 4.340 | Up | 3.49E-15 |
| BCAR4 | 3.738 | Up | 2.98E-28 |
| AL138789.1 | 3.543 | Up | 1.28E-49 |
| AL513123.1 | 3.203 | Up | 2.61E-08 |
| MIR3150BHG | 2.915 | Up | 1.65E-63 |
| LINC00536 | 2.775 | Up | 3.71E-50 |
| AC006262.3 | 2.536 | Up | 1.52E-04 |
| AL133467.1 | -2.265 | Down | 1.90E-55 |
| AC105398.1 | -2.330 | Down | 4.26E-16 |

Abbreviations: lncRNA: Long non-coding RNA.
