# Supplementary material for: Individualized Prediction of Survival by a 10-Long Non-coding RNA-Based Prognostic Model for Patients With Breast Cancer
Source: Front Oncol. 2020 Oct 19;10:515421. doi: 10.3389/fonc.2020.515421 (PMC7604500; doi:10.3389/fonc.2020.515421)
Supplement: Supplementary Table 4 — Clinical baseline characteristics of breast cancer patients in situ hybridization analysis. [file Table_4.DOCX]

**Supplementary Table 4.** Clinical baseline characteristics of breast cancer patients in *in situ* hybridization analysis

| **Characteristic** | **LHX1-DT expression** | | **Total (n=126)** | ***P* value** |
| --- | --- | --- | --- | --- |
|  | **Low (n=92)** | **High (n=34)** |  |  |
| **Age (years)** |  |  |  | 0.941 |
| <65 | 73 | 26 | 99 |  |
| ≥65 | 19 | 8 | 27 |  |
| **Tumor stage** |  |  |  | 0.593 |
| T1 | 39 | 11 | 50 |  |
| T2-T3 | 53 | 23 | 76 |  |
| **Lymph node metastasis** |  |  |  | 0.639 |
| N0 | 52 | 16 | 68 |  |
| N1-N3 | 40 | 18 | 58 |  |
| **TNM stage** |  |  |  | 0.873 |
| I-II | 64 | 22 | 86 |  |
| III | 28 | 12 | 40 |  |
| **ER status** |  |  |  | 0.390 |
| Negative | 22 | 14 | 36 |  |
| Positive | 67 | 20 | 87 |  |
| Not report | 3 | 0 | 3 |  |
| **PR status** |  |  |  | 0.232 |
| Negative | 36 | 21 | 57 |  |
| Positive | 54 | 13 | 67 |  |
| Not report | 2 | 0 | 2 |  |
| **HER2 status** |  |  |  | 0.261 |
| Negative | 35 | 20 | 55 |  |
| positive | 41 | 12 | 53 |  |
| Not report | 16 | 2 | 18 |  |

Abbreviations: ER, estrogen receptor; PR, progesterone receptor; HER2, human epithelial growth factor receptor 2.
